# FePO_4_ NPs Are an Efficient Nutritional Source for Plants: Combination of Nano-Material Properties and Metabolic Responses to Nutritional Deficiencies

**DOI:** 10.3389/fpls.2020.586470

**Published:** 2020-09-30

**Authors:** Davide Sega, Barbara Baldan, Anita Zamboni, Zeno Varanini

**Affiliations:** ^1^Department of Biotechnology, University of Verona, Verona, Italy; ^2^Department of Biology, University of Padua, Padua, Italy

**Keywords:** FePO_4_ nanoparticles, P, Fe, cucumber, maize

## Abstract

Phosphorous and iron are a macro- and micronutrient, respectively, whose low bioavailability can negatively affect crop productivity. There is ample evidence that the use of conventional P and Fe fertilizers has several environmental and economical disadvantages, but even though great expectations surround nanotechnology and its applications in the field of plant nutrition, little is known about the mechanisms underlying the uptake and use of these sub-micron particles (nanoparticles, NPs) by crop species. This work shows that cucumber and maize plants both use the nutrients borne by FePO_4_ NPs more efficiently than those supplied as bulk. However, morpho-physiological parameters and nutrient content analyses reveal that while cucumber plants (a *Strategy I* species with regard to Fe acquisition) mainly use these NPs as a source of P, maize (a *Strategy II* species) uses them preferentially for Fe. TEM analyses of cucumber root specimens revealed no cell internalization of the NPs. On the other hand, electron-dense nanometric structures were evident in proximity of the root epidermal cell walls of the NP-treated plants, which after ESEM/EDAX analyses can be reasonably identified as iron-oxyhydroxide. It appears that the nutritional interaction between roots and NPs is strongly influenced by species-specific metabolic responses.

## Introduction

Phosphorous (P) and iron (Fe) are a macro- and micronutrient, respectively, whose low bioavailability can seriously limit crop productivity (Barber, 1995). The application of fertilizers to correct P and Fe deficiency has a strong environmental and economic impact, given the low nutrient use efficiency (NUE) of the P-based fertilizers (around 10–20%) (Baligar et al., 2001) and the extreme leachability of Fe chelates. Fertilizers however are usually necessary, since P and Fe deficiency is estimated to occur in almost 65% and 30% of all arable land, respectively. It has been calculated that agriculture is responsible for 10% of all greenhouse gas emissions (EPA, 2017), hence it is essential to reduce its impact on the environment. Nanotechnology in this respect is extremely promising, and could be the key to more sustainable practices (Fu et al., 2019; Bartolucci et al., 2020). Nanofertilizers are classified into four groups: macronutrient nanofertilizers, micronutrient nanofertilizers, nanomaterial-enhanced fertilizers, and plant growth stimulating nanomaterials (Liu and Lal, 2016). Thanks to their high surface area to volume ratio, these compounds would appear to be more effective than conventional fertilizers in increasing NUE and reducing the amount of elements applied and released into the environment (Marchiol et al., 2020).

P is a macronutrient playing several key roles in plant biochemistry: it is a structural element of nucleic acids, and phospholipids, and is involved in vital reactions such as energy transfer, respiration, and photosynthesis (Hawkesford et al., 2012). Typical P-deficiency symptoms include reduced leaf expansion and a consequent increase in chlorophyll content and the reduction in the shoot/root ratio brought about by a major inhibition in shoot growth rather than root (Hawkesford et al., 2012). Plant roots can respond to P deficiency by exuding organic acids, H^+^ and phosphatases into the soil to enhance its acquisition (Chiou and Lin, 2011). Fe is the micronutrient taken up by plants in greatest amounts (Broadley et al., 2012), and is involved in a variety of metabolic processes such as respiration, photosynthesis, and chlorophyll biosynthesis (Broadley et al., 2012; Kobayashi and Nishizawa, 2012). Fe deficiency causes leaf chlorosis and negatively affects root elongation with an increase in the diameter of both apical root zones and root hairs (Hawkesford et al., 2012). Plants have evolved a variety of mechanisms to boost Fe acquisition and overcome its shortage. *Strategy I* species (non-grasses) respond to low Fe availability by extruding H^+^ into the rhizosphere thanks to the activity of plasma membrane (PM) H^+^-ATPases, reducing Fe(III) to Fe(II) by ferric-chelate reductase oxidase (FRO) and taking up Fe(II) by means of iron-regulated transporters (IRT) (Kobayashi and Nishizawa, 2012). Grass species on the other hand rely on *Strategy II*, consisting in the release of chelating agents (phytosiderophores, PS) through specific transporters (TOM) (Kobayashi and Nishizawa, 2012). These natural chelates have a high affinity for Fe(III) and the roots take up the Fe-PS complexes via YELLOW STRIPE transporters (YS) (Kobayashi and Nishizawa, 2012).

Investigations on the plant-soil system have generally highlighted a greater effectiveness of nano-scale P fertilizers with respect to conventional ones (Bala et al., 2014; Liu and Lal, 2014; Sharonova et al., 2015; Soliman et al., 2016; Taşkın et al., 2018). On the other hand, hydroponically-grown tomatoes treated for 48 h with either nano-hydroxyapatite or its bulk counterpart revealed no significant difference in P content and other parameters linked to plant metabolism (Marchiol et al., 2019). Fe oxide NPs (nFe_2_O_3_ and nFe_3_O_4_) have been the focus of much research: the literature describes a wide array of plant material and growth methods , and the effects observed depend on the conditions employed and the species analyzed (Marchiol et al., 2020). Only few of these investigations however compare these NPs with other, more conventional sources of Fe (*e.g.* FeCl_3_ and Fe-EDTA), either in pot (Palmqvist et al., 2017) or hydroponics experiments (Ghafariyan et al., 2013; Hu et al., 2017). In hydroponics-grown material, the effects of γ-Fe_2_O_3_ NPs on plant growth and oxidative stress were observed to depend on the concentration of the NPs (Hu et al., 2017). Furthermore, (Ghafariyan et al., 2013) showed that nFe_3_O_4_ NPs-treated soybean plants displayed a chlorophyll a to b ratio similar of that measured in chelate-treated ones. These authors also observed that the NPs can enter into and translocate inside the plant (Ghafariyan et al., 2013). Interestingly, the effects of Fe oxide NPs on plant growth and development are strongly linked to their size, as observed in hydroponics-grown tobacco plants treated with Fe_3_O_4_ (Alkhatib et al., 2019). Recent trials performed on hydroponic cultures also revealed that nanoscale Fe hydr(oxide) stabilized by humic compounds is a valid alternative to artificial chelates as a source of Fe (Kulikova et al., 2017).

In consequence of previous investigations (Sega et al., 2019), we decided to perform a morpho-physiological investigation on how FePO_4_ NPs are used as a source of nutrients by two plant species. The results reveal that this nano-sized material is more efficient than the bulk counterpart in delivering P and Fe, with performances sometimes similar to the positive controls, grown in the presence of the readily-available ionic forms. However, the response of the plants examined (*i.e.* cucumber and maize) was observed to depend on their specific metabolic adaptations to P and Fe nutritional deficiencies.

## Materials and Methods

### FePO_4_ NPs

The FePO_4_ NPs used in this study belong to the same batch used and thoroughly characterized in a previous work (Sega et al., 2019). In brief, citrate-capped FePO_4_ NPs were spheroidal and smaller than 20 nm, but could aggregate together with a size peak of 59 nm. About 90% of aggregates were smaller than 100 nm, and zeta potential was determined to be −45.0 ± 0.55 mV. Moreover, Fe/P molar ratio of the suspension was 1.055 and X-Ray Diffraction analysis showed the amorphous nature of FePO_4_ NPs.

### Plant Material and Growth Conditions

*Cucumis sativus* var. Viridis F1 hybrid seeds (Franchi Sementi S.p.A.) and *Zea mays* L. inbred line P0423 (Pioneer Hybrid Italia S.p.A.) were also grown as described by Sega et al. (2019). Cucumber seeds were germinated on paper towel moistened with 1 mM CaSO_4_ at 24°C in the dark. After 6 days, 6 seedlings per condition were transferred to 2-L pots containing aerated nutrient solution. Maize seeds were germinated on paper towel moistened with deionized water at 25°C in the dark. After 3 days, 6 seedlings per condition were transferred to 2-L pots containing aerated nutrient solution. Plants were grown at 24±2 °C under a 16/8 h light/dark photoperiod with light intensity of 200 to 250 μmol m^−2^ s^−1^ as PPFD (Photosynthetic Photon Flux Density) at the plants level. The complete nutrient solution (control) was modified in order to obtain the following conditions: plants grown in a complete nutrient solution (C); plants grown without P (**-**P), without Fe (**-**Fe), and in the absence of both P and Fe (**-**P**-**Fe); plants grown with FePO_4_ NPs as the source of P (**-**P+NPs), as the source of Fe (**-**Fe+NPs), and of both P and Fe (**-**P**-**Fe+NPs), plants grown with bulk FePO_4_ as the source of P (**-**P+b), as the source of Fe (**-**Fe+b), and of both P and Fe (**-**P**-**Fe+b). FePO_4_ NPs and bulk FePO_4_ were supplied at a final concentration of 100 μM (**Supplementary Figure S1**). The complete nutrient solution (C) had the following composition: 0.7 mM K_2_SO_4_, 2 mM Ca(NO_3_)_2_, 0.5 mM MgSO_4_, 0.1 mM KH_2_PO_4_, 0.1 mM KCl, 100 μM FeNaEDTA, 10 μM H_3_BO_3_, 0.5 μM MnSO_4_, 0.5 μM ZnSO_4_, 0.2 μM CuSO_4_, and 0.01 μM (NH_4_)_6_Mo_7_O_24_. Three independent growth and treatment experiments (biological replicates) with six plants each (technical replicates) were performed. The plants sampling occurred after 14 and 17 days of growth for cucumber and maize, respectively. At the sampling time, SPAD index was measured for all plants, while root apparatuses of three plants per pot were scanned for *WinRHIZO^TM^ analysis*. Three plants per pot were washed 5 times with deionized water (18.2 MΩ·cm at 25 °C) and dried at 60 °C for 72 hours, then weighted (dry weight) and processed for the determination of macro- and micronutrients.

### SPAD Index Measurement and Plants Sampling

SPAD index measurements and plant sampling were performed after 14 and 17 days of growth of the cucumber and maize seedlings, respectively. At these time points, the various treatment displayed visible differences brought about by their nutritional status. The SPAD index was determined by taking five measurements per leaf using a SPAD-502 Plus Chlorophyll Meter® (Konica Minolta). In cucumber plants, the measurements were taken on the first leaf of each plant which was the only fully expanded one. In the case of maize, the SPAD index was determined on all the leaves.

### Anthocyanin Quantification in Root Tissues

Frozen maize root tissues were homogenized with a mortar and pestle using liquid nitrogen. Anthocyanins were extracted from 300 mg of homogenate after the addition of 3 mL methanol acidified with 1% HCl. The mix was incubated for 4 hours in the dark at 4°C, and mixed every 30 minutes. The extracts thus obtained were centrifuged at 12000 rcf for 1 hour. Supernatant absorbance was measured with an Evolution 201 spectrophotometer (Thermo Scientific) at 530 and 657 nm. Anthocyanin content was determined as described by Mancinelli (Mancinelli, 1984) and expressed as µg of cyanidine-3-glucoside· gFW^−1^ (Wrolstad, 1976).

### WinRHIZO^TM^ Analysis

The root systems of three plants per pot were scanned with an Epson Perfection V700 scanner, and the images were analyzed with the WinRHIZO^TM^ software, 2015a Pro version (Regent Instruments Inc.), using the “root morphology” mode. This software analyses the digital images, estimating parameters such as total root length and root surface area, making it possible to estimate the effects of the treatments on root development.

### Determination of Macro- and Micronutrient Content in Plant Tissues

Dried samples were ground using a mortar and pestle, and approximately 10 to 20 mg of homogenized material was mineralized in a 3-ml TFM microsampling insert (Milestone Srl) using 250 μL of ultrapure grade HNO_3_ (Romil). The digestion was performed at 180 °C for 20 minutes in a StartD (Milestone Srl) microwave digestor. Three inserts were placed in a TFM 100-mL vessel with 11 mL of Milli-Q water and 1 mL of ultrapure grade H_2_O_2_ (69%. Fisher Scientific). The digested samples were diluted to 2% HNO_3_ with ultra-pure grade water (18.2 MΩ·cm at 25 °C), and analyzed using an Agilent 7500ce ICP-MS detection system (Agilent technologies). Calibration curves were obtained by diluting a custom-made multielement standard (Romil LTD), with the a stock solution containing K (20,000 ppm), Ca (10,000 ppm), Mg and P (2,000 ppm), Na (400 ppm), Fe (50 ppm) Mn (40 ppm), B and Zn (20 ppm), Cu (5 ppm), Co, Mo, and Se (1 ppm). Measurement accuracy and matrix effect errors were checked using a standard reference material (NIST 1515 Apple leaves), which was digested and analyzed in the same way as the samples. Concentrations of elements that could not be determined in the reference material within a range of ± 10 % of the declared value were not further processed and are not reported in Results section.

### TEM Analysis of Cucumber Roots

Portions of tertiary roots harvested from cucumber plants grown with FePO_4_ NPs as P source (−P+NPs) were fixed in a 1.5% glutaraldehyde solution for 24 hours at 4°C in the dark. The root samples were then rinsed three times with a 0.1 M cacodylate buffer, pH 7.0, and post-fixed for 2 h with 1% (w/v) osmium tetroxide in a 0.1 M cacodylate buffer (pH 7.0) in the dark. The samples were once again rinsed three times with a 0.1 M cacodylate buffer, pH 7.0, dehydrated in graded series of ethanol, and embedded in araldite resin. Ultra-thin (70 nm) sections were obtained with a Reichert-Jung ultramicrotome (Leica Biosystems, Wetzlar, Germany) and mounted on uncoated copper grids for observation with a Tecnai G2 (FEI) Transmission Electron Microscope (TEM) operating at 120 kV.

### ESEM and EDAX Analyses of Cucumber Roots

Portions of the roots of cucumber plants grown with FePO_4_ NPs as P source (**-**P+NPs) were rinsed three times in deionized water, dried gently with blotting paper and viewed with a Quanta 200 (FEI) Environmental Scanning Electron Microscope (ESEM) operating at 20 kV, in order to detect electron-dense crusts and analyze them by Energy-Dispersive X-ray Spectroscopy (EDAX).

## Results

In order to test the effectiveness of FePO_4_ NPs (NPs) as a source of P and Fe for plant nutrition, we performed experiments on two hydroponically-grown crop species with different response strategies to Fe shortage: cucumber (*Strategy I*) and maize (*Strategy II*). The rationale behind choosing both a *Strategy I* and a *Strategy II* plant is that the NPs employed for these trials bear both Fe and P and that *Strategy I* species share several common mechanisms to acquire these two elements (Watt and Evans, 1999; Tomasi et al., 2009). Details of the experimental design, devised by Sega et al. (2019), are reported in **Supplementary Figure S1**.

### Effects of NP Treatment on Morpho-Physiological Parameters

**Figure 1:**
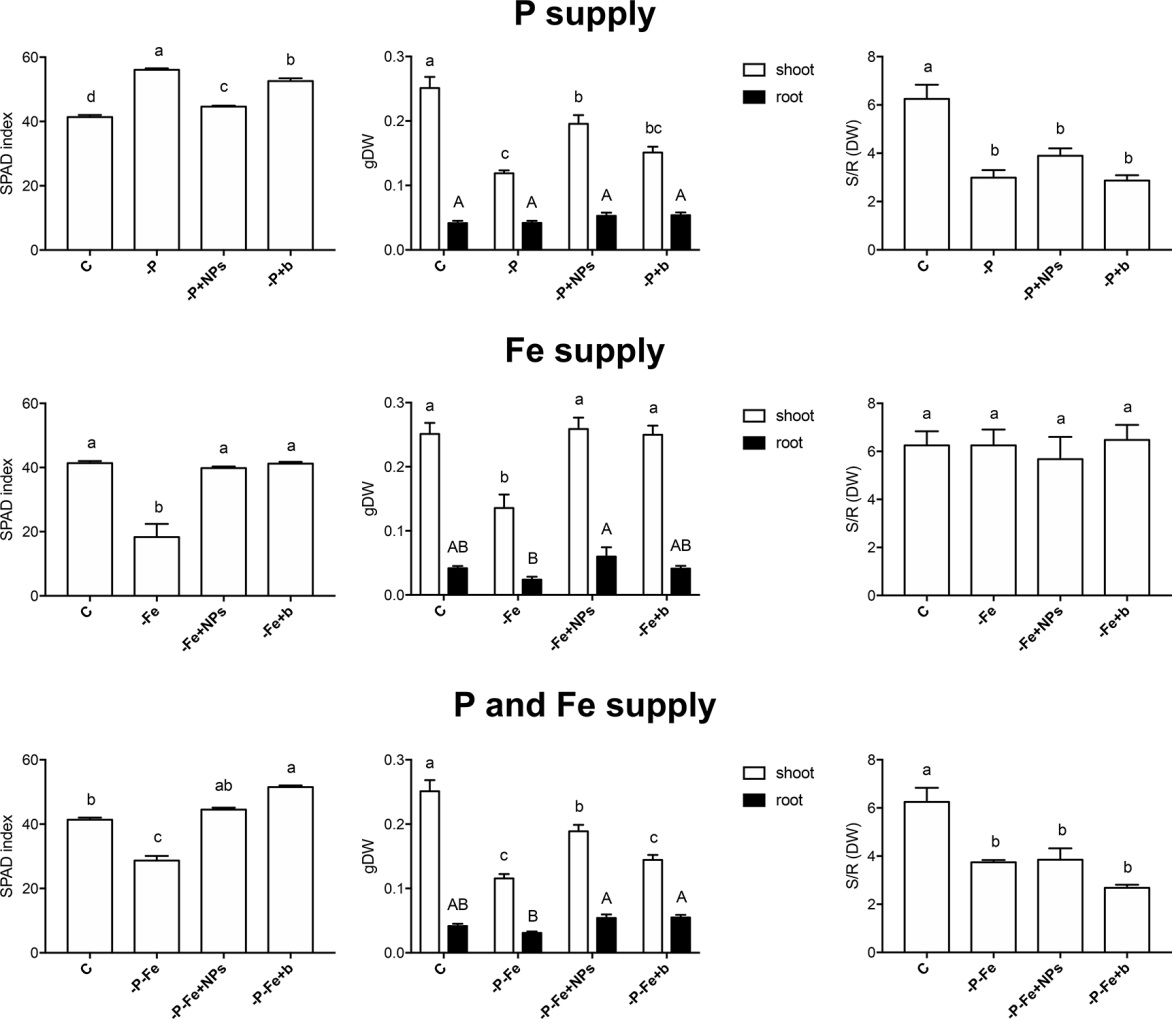
SPAD index, shoot and root dry weight and shoot/root dry weight ratio of cucumber plants at the end of the treatment (14 days). Plants treated with NPs as the source of P, Fe, or both nutrients (-P+NPs, -Fe+NPs, and -P-Fe+NPs, respectively) were compared with positive controls (C), plants grown in the absence of the respective nutrients (-P, -Fe and -P-Fe) and with bulk FePO_4_ as the source of P, Fe, or both. Data are expressed as means ± *SE* (*n*= 9, three independent experiments with three plants each; one-way ANOVA with Tukey’s post hoc test, *p* < 0.05, significant differences are indicated by different letters).

**Figure 2:**
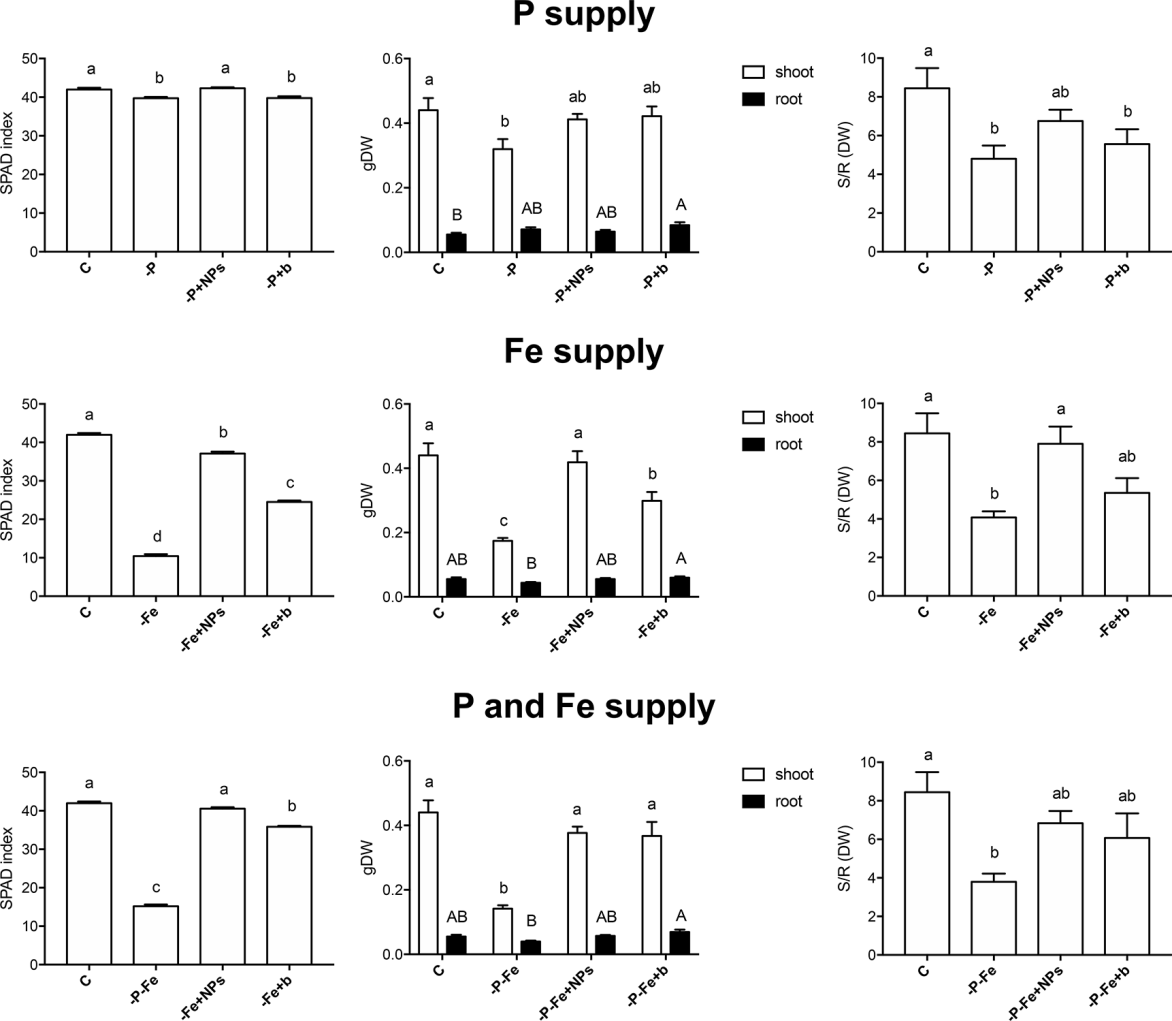
SPAD index, shoot and root dry weight and shoot/root dry weight ratio of maize plants at the end of the treatment (17 days). Plants treated with NPs as the source of P, Fe, or both nutrients (-P+NPs, -Fe+NPs, and -P-Fe+NPs, respectively) were compared with positive controls (C), plants grown in the absence of the respective nutrients (-P, -Fe, and -P-Fe) or with bulk FePO_4_ as the source of P, Fe, or both. Data are expressed as means ± SE (*n* = 9, three independent experiments with three plants each; one-way ANOVA with Tukey’s post hoc test, *p* < 0.05, significant differences are indicated by different letters).

The ability of NPs to provide P and Fe was evaluated at the end of the growth period (14 and 17 days for cucumber and maize plants, respectively) by determining leaf SPAD index, shoot and root dry weight, and shoot/root ratio (**Figures 1**, **2**, **Supplementary Figures S2**–**S4**). As regards P nutrition in cucumber, the plants treated with NPs displayed SPAD values close to those of positive controls. Furthermore, they showed a biomass greater than both their negative controls and the bulk-treated plants (**Figure 1**). However, **-**P, **-**P+NPs, and **-**P+b plants all displayed a lower shoot/root ratio than the controls (C) (**Figure 1**).

As regards maize, the SPAD index values of the negative controls were lower than both the positive controls and NP-treated plants (**Figure 2**). On the other hand, the negative controls accumulated anthocyanins in their roots (**Supplementary Figure S5**) and the leaves of some plants exhibited sporadic signs of P deficiency (**Supplementary Figure S6**) (Calderón-Vázquez et al., 2011; Henry et al., 2012). Interestingly, the root anthocyanin contents of NP-treated plants were similar to those of the positive controls. Altogether these data suggest the onset of P-deficient conditions in **-**P and **-**P+b plants and the positive effect exerted by NPs as a source of P.

Conversely, the effects of NPs on Fe nutrition were unlike those described for P. Fe-treated cucumber plants displayed no significant difference in their SPAD index, independently of the source of Fe employed (**Figure 1**). When grown in the absence of Fe (**-**Fe), this plant species displayed—as expected—the lowest SPAD index values and visible symptoms of chlorosis (Broadley et al., 2012). Furthermore, no significant differences were recorded in the shoot to root dry biomass ratio (**Figure 1**). Maize plants on the other hand displayed a different reaction: application of NPs gave rise to significantly higher SPAD indexes than those observed in bulk-treated plants, comparable to those of their positive control (**Figure 2**). Likewise, the shoot biomass and the shoot to root dry weight ratio were also similar to those of the controls.

Even when P and Fe were considered simultaneously (**-**P**-**Fe), the effects seemed to be species-specific. Data relative to the SPAD index suggest that NPs were a better source of these two elements than the bulk form, given that the plants, and maize in particular, displayed values similar to their positive controls (**Figures 1**, **2**). The lower SPAD indexes measured in the negative controls (**-**P**-**Fe) of both cucumber and maize seem to indicate that Fe deficiency has a greater impact on leaf physiology than the lack of P. Moreover, the bulk form of FePO_4_ proved to be a less efficient source of P for cucumber plants and of Fe for maize. In the former species, bulk-treated plants exhibited the highest SPAD values, a sign of P deficiency, whilst the lowest ones, more typical of Fe shortage, were exhibited in the latter one. These results were also confirmed by shoot/root ratio values. In cucumber, the negative controls, as well as both NP- and bulk-treated plants all displayed significantly lower values of this parameter. In maize however, no significant differences emerged between the plants treated with either form of FePO_4_ and their positive control.

**Figure 3:**
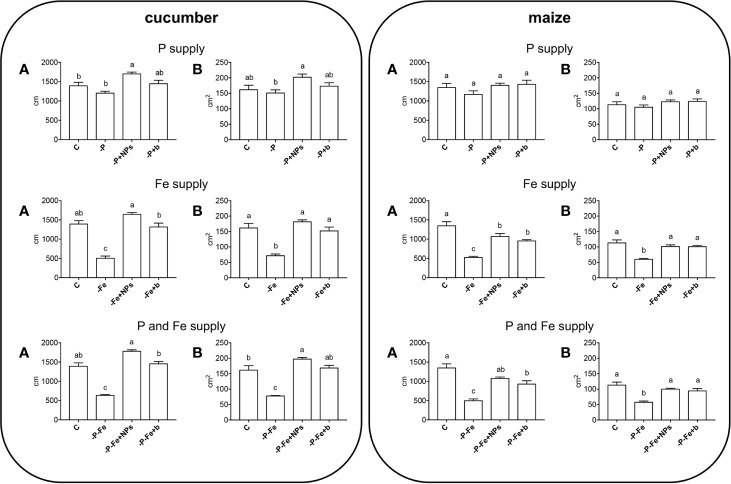
Total root length **(A)** and surface area **(B)** of cucumber and maize plants sampled after 14 and 17 days of growth, respectively. Plants treated with NPs as the source of P, Fe, or both (-P+NPs, -Fe+NPs, and -P-Fe+NPs, respectively) were compared with positive controls (C), plants grown in the absence of the respective nutrients (-P, -Fe, and -P-Fe) or with bulk FePO_4_ as the source of P, Fe or both. Data are expressed as means ± SE (*n* = 9, three independent experiments with three plants each; one-way ANOVA with Tukey’s post hoc test, *p* < 0.05, significant differences are indicated by different letters).

As regards root morphology, **Figure 3** shows that NPs exerted a positive effect on the length of cucumber roots, which were more developed than both bulk-treated plants and the positive controls under all the nutritional conditions tested (P, Fe, P, and Fe supply), although it should be stressed that no statistically significant difference emerged when measuring root dry biomass (**Figure 1**). Root elongation appears to be linked to the species, since it was less evident in maize.

### Effects of NPs on Macro- and Micronutrient Contents in Root and Shoot Tissues

The treatments employed changed the tissue concentrations of not only P and Fe, but also other macro- and micronutrients (**Tables 1**, **2**). In general, in both plant species, the greatest number of significant differences was displayed in the treatment involving Fe supply.

**Table 1:** Macro- and micronutrient concentration in cucumber root and shoot tissues.

	Root				Shoot			
P supply								
	C	-P	-P+NPs	-P+b	C	-P	-P+NPs	-P+b
Ca (mg g^−1^ DW)	7.65 ± 0.55	8.06 ± 0.37	7.81 ± 0.52	7.64 ± 0.47	**61.98 ± 1.62^a^**	**56.77 ± 3.67^ab^**	**55.62 ± 1.38^ab^**	**50.43 ± 1.04^b^**
K (mg g^−1^ DW)	101.02 ± 4.71	92.52 ± 1.42	99.40 ± 5.48	102.72 ± 7.50	**54.19 ± 2.38^ab^**	**34.27 ± 2.93^c^**	**56.84 ± 3.03^a^**	**44.77 ± 1.87^b^**
Mg (mg g^−1^ DW)	2.77 ± 0.14	2.68 ± 0.08	2.78 ± 0.18	2.95 ± 0.17	6.76 ± 0.13	7.18 ± 0.59	6.77 ± 0.12	6.98 ± 0.15
P (mg g^−1^ DW)	**8.22 ± 0.29^a^**	**2.00 ± 0.06^c^**	**6.20 ± 0.61^b^**	**4.69 ± 0.61^b^**	**14.37 ± 0.41^a^**	**1.72 ± 0.12^c^**	**6.26 ± 0.48^b^**	**2.53 ± 0.05^c^**
Cu (*μ*g g^−1^ DW)	12.72 ± 2.42	13.05 ± 1.40	15.44 ± 1.72	14.03 ± 1.07	12.64 ± 0.57	12.98 ± 0.65	13.72 ± 0.33	12.63 ± 0.51
Fe (*μ*g g^−1^ DW)	**1188.50 ± 74.52^c^**	**1233.72 ± 81.82^c^**	**9463.41 ± 1343.53^a^**	**5971.71 ± 786.23^b^**	182.29 ± 12.99	211.52 ± 24.80	204.87 ± 35.62	255.61 ± 18.47
Mn (*μ*g g^−1^ DW)	**16.97 ± 1.74^b^**	**80.07 ± 7.32^a^**	**25.76 ± 5.89^b^**	**35.16 ± 4.41^b^**	**44.43 ± 2.75^c^**	**86.80 ± 4.64^a^**	**43.55 ± 1.53^c^**	**62.77 ± 1.84^b^**
Zn (*μ*g g^−1^ DW)	**132.47 ± 14.53^a^**	**70.83 ± 4.69^b^**	**119.49 ± 20.81^ab^**	**94.53 ± 12.79^ab^**	**100.51 ± 4.36^a^**	**66.80 ± 5.34^bc^**	**81.42 ± 3.20^b^**	**65.57 ± 2.61^c^**
**Fe supply**								
	**C**	**-Fe**	**-Fe+NPs**	**-Fe+b**	**C**	**-Fe**	**-Fe+NPs**	**-Fe+b**
Ca (mg g^−1^ DW)	7.65±0.55	9.60 ± 0.93	8.03 ± 0.48	8.41 ± 0.44	**61.98 ± 1.62^b^**	**67.86 ± 1.40^a^**	**64.95 ± 1.17^ab^**	**67.23 ± 1.56^ab^**
K (mg g^−1^ DW)	**101.02 ± 4.71 ab**	**77.62 ± 2.56^b^**	**113.07 ± 7.06^a^**	**104.64 ± 10.35^ab^**	**54.19 ± 2.38^ab^**	**62.42 ± 4.61^a^**	**52.31 ± 2.14^ab^**	**51.24 ± 1.51^b^**
Mg (mg g^−1^ DW)	2.77 ± 0.14	2.46 ± 0.16	2.80 ± 0.12	2.72 ± 0.21	**6.76 ± 0.13^c^**	**14.07 ± 0.53^a^**	**7.57 ± 0.11^bc^**	**9.45 ± 0.20^b^**
P (mg g^−1^ DW)	8.22 ± 0.29	6.61 ± 0.32	9.42 ± 1.01	8.87 ± 1.42	**14.37 ± 0.41^a^**	**11.03 ± 0.38^b^**	**14.31 ± 0.19^a^**	**14.83 ± 0.76^a^**
Cu (*μ*g g^−1^ DW)	**12.72 ± 2.42^c^**	**399.18 ± 65.25^a^**	**53.88 ± 7.12^bc^**	**175.31 ± 21.69^b^**	**12.64 ± 0.57^a^**	**27.45 ± 2.09^b^**	**15.11 ± 0.37^a^**	**16.47 ± 0.53^a^**
Fe (*μ*g g^−1^ DW)	**1188.50 ± 74.52^c^**	**114.23 ± 24.23^c^**	**8109.19 ± 904.02^a^**	**3069.75 ± 686.82^b^**	**182.29 ± 12.99^a^**	**87.33 ± 9.90^b^**	**201.87 ± 21.10^a^**	**166.81 ± 17.53^a^**
Mn (*μ*g g^−1^ DW)	**16.97 ± 1.74^c^**	**48.92 ± 8.82^b^**	**35.39 ± 4.47^bc^**	**86.80 ± 13.19^a^**	**44.43 ± 2.75^c^**	**257.07 ± 25.42^a^**	**72.70 ± 5.06^c^**	**132.71 ± 6.24^a^**
Zn (*μ*g g^−1^ DW)	**132.47 ± 14.53^b^**	**543.91 ± 80.93^a^**	**179.06 ± 20.10^b^**	**378.81 ± 36.62^a^**	**100.51 ± 4.36^b^**	**207.43 ± 15.59^a^**	**111.40 ± 3.00^b^**	**132.74 ± 10.80^b^**
**P and Fe supply**								
	**C**	**-P-Fe**	**-P-Fe+NPs**	**-P-Fe+b**	**C**	**-P-Fe**	**-P-Fe+NPs**	**-P-Fe+b**
Ca (mg g^−1^ DW)	**7.65 ± 0.55^b^**	**10.28 ± 0.94^a^**	**8.07 ± 0.56^ab^**	**7.07 ± 0.51^a^**	61.98 ± 1.62	60.98 ± 1.53	58.62 ± 2.47	64.72 ± 2.29
K (mg g^−1^ DW)	101.02 ± 4.71	104.91 ± 15.14	98.24 ± 5.43	95.19 ± 8.61	**54.19 ± 2.38^a^**	**48.83 ± 1.63^a^**	**51.55 ± 3.94^a^**	**35.59 ± 1.64^b^**
Mg (mg g^−1^ DW)	2.77 ± 0.14	2.88 ± 0.52	2.71 ± 0.12	2.77 ± 0.24	**6.76 ± 0.13^b^**	**13.47 ± 0.27^a^**	**6.98 ± 0.35^b^**	**7.20 ± 0.16^b^**
P (mg g^−1^ DW)	**8.22 ± 0.29^a^**	**5.09 ± 1.10^bc^**	**6.84 ± 0.45^ab^**	**3.61 ± 0.33^c^**	**14.37 ± 0.41^a^**	**2.33 ± 0.06^c^**	**4.51 ± 0.31^b^**	**2.13 ± 0.06^c^**
Cu (*μ*g g^−1^ DW)	**12.72 ± 2.42^b^**	**236.41 ± 39.77^a^**	**62.27 ± 9.74^b^**	**87.16 ± 13.13^b^**	**12.64 ± 0.57^c^**	**29.40 ± 0.65^a^**	**16.97 ± 0.35^b^**	**17.64 ± 0.28^b^**
Fe (*μ*g g^−1^ DW)	**1188.50 ± 74.52^bc^**	**106.82 ± 11.31^c^**	**8277.52 ± 1223.75^a^**	**2917.63 ± 440.39^b^**	**182.29 ± 12.99^a^**	**92.56 ± 11.50^c^**	**137.60 ± 10.97^b^**	**125.19 ± 8.18^bc^**
Mn (*μ*g g^−1^ DW)	**16.97 ± 1.74^b^**	**116.95 ± 20.61^a^**	**31.04 ± 3.02^b^**	**88.13 ± 6.16^a^**	**44.43 ± 2.75^c^**	**304.66 ± 18.83^a^**	**75.20 ± 5.28^c^**	**114.34 ± 4.96^b^**
Zn (*μ*g g^−1^ DW)	**132.47 ± 14.53^b^**	**481.00 ± 68.19^a^**	**181.17 ± 23.49^b^**	**200.62 ± 17.80^b^**	**100.51 ± 4.36^b^**	**193.07 ± 12.57^a^**	**87.27 ± 4.27^b^**	**92.33 ± 3.35^b^**

Data of plants treated for 14 days with NPs as the source of either P, Fe or both nutrients (-P+NPs, -Fe+NPs and -P-Fe+NPs) were compared to those of the positive controls (C), the respective negative controls, i.e. plants grown in the absence of these nutrients (-P, -Fe and -P-Fe) and ones grown with bulk FePO_4_ as the source of Fe, P or both. Data are expressed as mean ± SE (n= 9, three independent experiments with three plants each; one-way ANOVA with Tukey’s post hoc test, p < 0.05, significant data in bold, significant differences are indicated by different letters).

**Table 2:** Macro- and micronutrient concentration in maize root and shoot tissues.

	Root				Shoot			
P supply								
	C	-P	-P+NPs	-P+b	C	-P	-P+NPs	-P+b
Ca (mg g^−1^ DW)	8.19 ± 0.48	7.39 ± 0.43	6.67 ± 0.46	6.76 ± 0.29	**6.50 ± 0.18^ab^**	**6.15 ± 0.17^b^**	**7.12 ± 0.22^a^**	**6.40 ± 0.28^ab^**
K (mg g^−1^ DW)	**46.12 ± 1.99^a^**	**40.20 ± 2.74^ab^**	**37.30 ± 1.47^b^**	**37.06 ± 1.39^b^**	92.05 ± 3.23	81.65 ± 2.78	82.60 ± 1.81	84.37 ± 3.92
Mg (mg g^−1^ DW)	**2.69 ± 0.15^b^**	**2.94 ± 0.15^ab^**	**2.63 ± 0.12^b^**	**3.35 ± 0.13^a^**	**2.92 ± 0.06^a^**	**2.54 ± 0.07^bc^**	**2.74 ± 0.08^ab^**	**2.40 ± 0.07^c^**
P (mg g^−1^ DW)	**3.74 ± 0.26^a^**	**1.37 ± 0.11^c^**	**2.94 ± 0.18^b^**	**1.65 ± 0.15^c^**	**11.32 ± 0.73^a^**	**2.09 ± 0.13^b^**	**3.36 ± 0.15^b^**	**2.13 ± 0.15^b^**
Cu (*μ*g g^−1^ DW)	**28.10 ± 6.69^a^**	**14.62 ± 1.09^ab^**	**13.06 ± 0.54^b^**	**19.46 ± 2.20^ab^**	11.27 ± 1.12	9.90 ± 0.60	8.95 ± 0.26	9.99 ± 0.93
Fe (*μ*g g^−1^ DW)	**1030.85 ± 144.60^b^**	**810.06 ± 54.28^b^**	**6197.59 ± 448.32^a^**	**1883.75 ± 331.31^b^**	**134.03 ± 4.16^b^**	**148.11 ± 8.94^b^**	**251.35 ± 21.96^a^**	**176.43 ± 21.84^b^**
Mn (*μ*g g^−1^ DW)	**349.74 ± 29.38^a^**	**250.25 ± 15.09^b^**	**253.65 ± 14.32^b^**	**238.63 ± 21.65^b^**	55.22 ± 3.15	48.17 ± 3.54	47.71 ± 1.66	43.84 ± 2.71
Zn (*μ*g g^−1^ DW)	158.16 ± 8.04	205.76 ± 12.99	193.58 ± 19.68	196.27 ± 21.04	75.53 ± 4.47	78.54 ± 5.38	75.20 ± 3.76	75.51 ± 6.46
**Fe supply**								
	**C**	**-Fe**	**-Fe+NPs**	**-Fe+b**	**C**	**-Fe**	**-Fe+NPs**	**-Fe+b**
Ca (mg g^−1^ DW)	8.19 ±0.48	8.18 ±0.45	8.84 ±0.38	8.87 ±0.37	**6.50 ± 0.18^b^**	**8.84 ± 0.60^a^**	**6.24 ± 0.20^b^**	**6.83 ± 0.29^b^**
K (mg g^−1^ DW)	46.12 ± 1.99	50.40 ± 2.63	46.03 ± 1.85	47.85 ± 1.70	92.05 ± 3.23	87.84 ± 3.92	87.84 ± 3.27	86.72 ± 3.34
Mg (mg g^−1^ DW)	**2.69 ± 0.15^c^**	**4.52 ± 0.20^a^**	**3.39 ± 0.10^b^**	**4.46 ± 0.10^a^**	**2.92 ± 0.06^b^**	**3.59 ± 0.16^a^**	**3.03 ± 0.10^b^**	**3.31 ± 0.13^ab^**
P (mg g^−1^ DW)	**3.74 ± 0.26^b^**	**5.25 ± 0.35^a^**	**4.15 ± 0.19^ab^**	**4.60 ± 0.38^ab^**	**11.32 ± 0.73^b^**	**17.01 ± 0.98^a^**	**9.65 ± 0.55^b^**	**11.44 ± 0.62^b^**
Cu (*μ*g g^−1^ DW)	**28.10 ± 6.69^d^**	**235.83 ± 9.04^a^**	**80.94 ± 4.82^c^**	**147.63 ± 7.35^b^**	**11.27 ± 1.12^c^**	**25.19 ± 1.44^a^**	**14.97 ± 0.62^c^**	**20.97 ± 0.97^b^**
Fe (*μ*g g^−1^ DW)	**1030.85 ± 144.60^b^**	**65.33 ± 13.84^c^**	**2263.43 ± 254.85^a^**	**350.90 ± 42.26^c^**	**134.03 ± 4.16^a^**	**54.64 ± 4.87^d^**	**97.47 ± 3.96^b^**	**76.29 ± 4.86^c^**
Mn (*μ*g g^−1^ DW)	**349.74 ± 29.38^a^**	**230.10 ± 10.36^b^**	**208.75 ± 12.01^b^**	**193.16 ± 12.13^b^**	**55.22 ± 3.15^c^**	**133.42 ± 7.53^a^**	**59.61 ± 2.99^c^**	**86.05 ± 3.31^b^**
Zn (*μ*g g^−1^ DW)	**158.16 ± 8.04^b^**	**276.92 ± 15.65^a^**	**208.19 ± 13.95^b^**	**190.14 ± 23.71^b^**	**75.53 ± 4.47^c^**	**302.18 ± 27.66^a^**	**107.05 ± 5.68^c^**	**171.09 ± 9.67^b^**
**P and Fe supply**								
	**C**	**-P-Fe**	**-P-Fe+NPs**	**-P-Fe+b**	**C**	**-P-Fe**	**-P-Fe+NPs**	**-P-Fe+b**
Ca (mg g^−1^ DW)	8.19 ± 0.48	7.07 ± 0.46	8.09 ± 0.31	7.34 ±0.39	**6.50 ± 0.18^b^**	**7.72 ± 0.38^a^**	**5.95 ± 0.19^b^**	**5.76 ± 0.23^b^**
K (mg g^−1^ DW)	46.12 ± 1.99	48.73 ± 2.02	43.75 ± 2.05	40.97 ± 1.81	92.05 ± 3.23	90.45 ± 3.66	80.82 ± 3.32	79.07 ± 5.22
Mg (mg g^−1^ DW)	**2.69 ± 0.15^b^**	**4.34 ± 0.28^a^**	**3.28 ± 0.11^b^**	**3.25 ± 0.13^b^**	**2.92 ± 0.06^b^**	**3.43 ± 0.15^a^**	**2.52 ± 0.07^c^**	**2.42 ± 0.08^c^**
P (mg g^−1^ DW)	**3.74 ± 0.26^a^**	**1.65 ± 0.12^c^**	**2.39 ± 0.09^b^**	**1.64 ± 0.13^c^**	**11.32 ± 0.73^a^**	**3.87 ± 0.20^b^**	**3.30 ± 0.17^bc^**	**2.02 ± 0.21^c^**
Cu (*μ*g g^−1^ DW)	**28.10 ± 6.69^d^**	**243.98 ± 10.87^a^**	**64.12 ± 2.75^c^**	**121.94 ± 5.65^b^**	**11.27 ± 1.12^b^**	**25.81 ± 2.32 a**	**13.18 ± 0.42 b**	**13.10 ± 0.54^b^**
Fe (*μ*g g^−1^ DW)	**1030.85 ± 144.60^b^**	**138.17 ± 54.18^c^**	**3004.17 ± 172.49^a^**	**743.33 ± 115.50^b^**	**134.03 ± 4.16^a^**	**56.56 ± 7.00^c^**	**120.74 ± 18.66^ab^**	**85.36 ± 2.27^bc^**
Mn (*μ*g g^−1^ DW)	**349.74 ± 29.38^a^**	**229.52 ± 11.61^b^**	**191.04 ± 9.20^b^**	**209.45 ± 10.21^b^**	**55.22 ± 3.15^b^**	**153.72 ± 9.03^a^**	**57.20 ± 4.63^b^**	**59.18 ± 3.55^b^**
Zn (*μ*g g^−1^ DW)	**158.16 ± 8.04^c^**	**267.23 ± 20.79^a^**	**236.70 ± 17.28^ab^**	**191.99 ± 11.21^bc^**	**75.53 ± 4.47^c^**	**316.29 ± 17.78^a^**	**104.22 ± 3.36^bc^**	**114.36 ± 3.24^b^**

Data of plants treated for 17 days with NPs as the source of either P, Fe, or both nutrients (-P+NPs, -Fe+NPs, and -P-Fe+NPs) were compared to those of the positive controls (C), the respective negative controls, i.e. plants grown in the absence of these nutrients (-P, -Fe, and -P-Fe) and ones grown with bulk FePO_4_ as the source of Fe, P or both. Data are expressed as mean ± SE (n= 9, three independent experiments with three plants each; one-way ANOVA with Tukey’s post hoc test, p < 0.05, significant data in bold, significant differences are indicated by different letters).

With regard to P nutrition, **Table 1** shows that in the shoot of cucumber plants treated with NPs the levels of this element were more than double those found in bulk-treated or negative control plants, but lower than those of the positive controls. Similar results were observed when NPs were used as the source of both P and Fe. In maize too, NP-treated plants had a higher P concentration than that of **-**P and **-**P+b plants, although in the shoots, the difference was not statistically significant (**Table 2**). As regards Fe nutrition, the treatment with NPs strongly affected the concentration of this element in the root tissues of both species under all the nutritional conditions analyzed (**Tables 1**, **2**). The application of these compounds also increased Fe levels in the shoots of maize and, to a much lesser extent, cucumber plants. The pattern of total P and Fe accumulation in the shoots is shown in **Supplementary Table S1**. In cucumber shoots, the total amount of P supplied in NP form was significantly higher (about three times) than that of plants treated with its bulk counterpart. In the shoots of maize however, NP treatment determined much higher levels of total Fe than those measured in bulk-treated plants (**Supplementary Table S2**).

As regards divalent cations, the shoots of cucumber plants grown with NPs as a source of Fe had lower levels of Mg than those observed in **-**Fe and **-**Fe+b plants, with values comparable to the positive controls (**Table 1**). Although less evident, a similar trend was also displayed by maize plants (**Table 2**). Furthermore, the tissue concentrations of Cu, Mn, and Zn were significantly lower in both positive controls and NP-treated plants than in those treated with bulk or growing in the absence of Fe (−Fe). This was particularly evident in cucumber roots and maize shoot (**Tables 1**, **2**).

### TEM and ESEM Observation and EDAX Analysis of Cucumber Plants Roots Treated With FePO_4_ NPs

**Figure 4:**
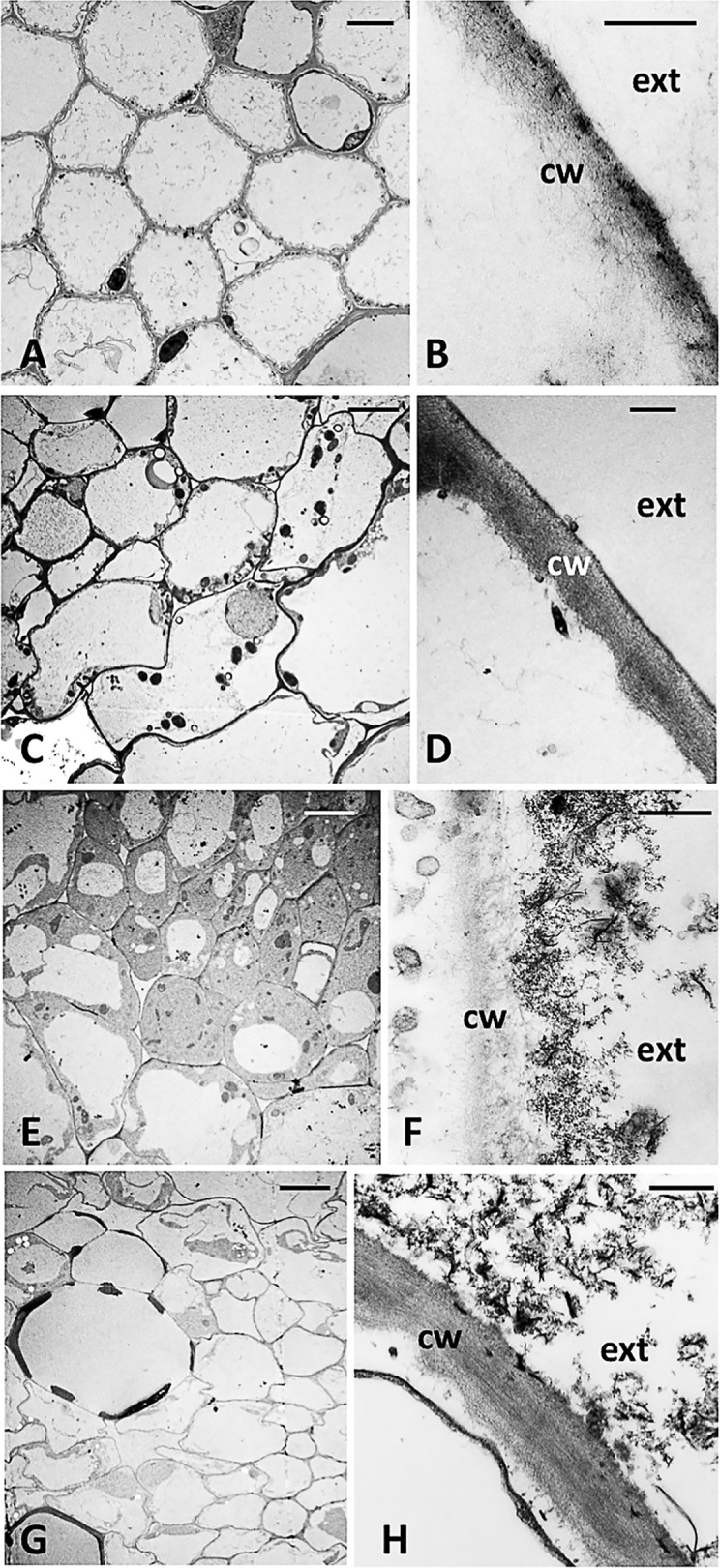
Cross-section TEM images of a tertiary root of a cucumber plant grown **(A, B)**: without P (-P), **(C, D)**: without P and Fe (-P-Fe), **(E, F)**: with FePO_4_ NPs as the source of P and **(G, H)**, with FePO_4_ NPs as the source of P and Fe; cw, cell wall; ext, external side. Bars A, C, E, G: 5 µm; B, D, F, H: 500 nm.

Cucumber plants grown for 14 days in the presence of NPs as the source of either P alone (−P+NPs) or of both P and Fe (**-**P**-**Fe+NPs) displayed a marked orange staining of their root system (**Supplementary Figures S3**, **S7**). To shed light on this aspect, less evident in maize, and verify whether NPs could enter into the cells, a TEM analysis was carried out on cross-sections of the tertiary roots of **-**P+NPs cucumber plants (**Figure 4**). As shown in the figures, no NPs were detected inside the root cells of plants treated with these compounds (**-**P+NPs and **-**P**-**Fe+NPs; **Figures 4E, G**), nor in the corresponding negative controls (**-**P and **-**P**-**Fe; **Figures 4A, C**). Interestingly, electron-dense nanometric structures, some of which lath-shaped, were evident on the outer side of the root epidermal cell walls of the NP-treated plants (**Figures 4F, H**). These structures were absent on the epidermal cell walls of both **-**P (**Figure 4B**) and **-**P**-**Fe plants (**Figure 4D**). In the former roots however, a thin coat of electron-dense material was observed, embedded in the more external layer of the cell wall. TEM analysis was also performed on the roots treated with NPs as source of Fe, which were whiter in color (**Supplementary Figure S3**). Scanning revealed that the shape of the electron-dense structures present in these **-**Fe+NPs specimens differed extensively from those observed in the epidermal cell walls of both **-**P+NPs and **-**P**-**Fe+NPs roots (**Supplementary Figure S8**). Interestingly, the outline of these structures was very similar to that of NPs aggregates *per se* (Sega et al., 2019).

**Figure 5:**
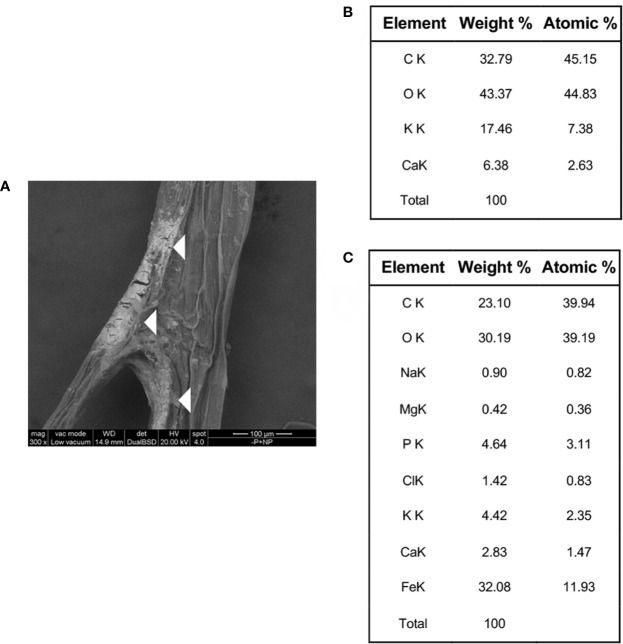
ESEM image and EDAX analysis of roots of a cucumber plant grown with FePO_4_ NPs as P source (−P+NPs). ESEM image of root surface **(A)**, with white triangles indicating the electron-dense crusts; weight and atomic percentage values resulting from EDAX analysis performed on a non-electron dense portion of the root surface **(B)**; weight and atomic percentage values resulting from EDAX analysis performed on an electron-dense portion of the root surface **(C)**.

To investigate the chemical nature of the orange staining, ESEM observations and EDAX analysis were carried out on portions of roots of plants grown with FePO_4_ NPs as P source. Electron-dense crusts were visible on the root surface (**Figure 5A**). EDAX scanning was performed on both the non-electron-dense and electron-dense areas of the root surface (**Supplementary Data set S1**). Analyses on the latter zone revealed an excess of Fe with respect to P, with a ratio between the two elements of 3.84 (**Figure 5C**). This value is much higher than the 1:1 ratio measured in NPs (Sega et al., 2019). Neither element was detected in the non-electron-dense zone (**Figure 5B**). ESEM observations and EDAX analyses showed that the Fe/P ratio was also greater than 1 on the colored surface of the roots of **-**P**-**Fe+NPs plants (**Supplementary Figure S3**, **Supplementary Data set S3**), suggesting that once again, there was a greater accumulation of Fe than P. In the plants where NPs were used as Fe source (**-**Fe+NPs) the analyses revealed a roughly 1:1 Fe to P ratio on their white roots (**Supplementary Figure S3**, **Supplementary Data set S2**).

## Discussion

The idea of using nano-scale fertilizers for crop nutrition has opened a field of research on their potential effects on plant growth (Marchiol et al., 2020). Most of these investigations however fail to take in account the possibility of using these compounds as a source of more than one nutrient. Given the results of previous investigations (Sega et al., 2019), we decided to focus in greater detail on the effects of FePO_4_ NPs, which in theory should be able to deliver P and Fe simultaneously. We also took into account that plants possess two different mechanisms to acquire Fe from soil and respond to conditions of deficiency, *i.e. Strategy I* and *Strategy II* (Kobayashi and Nishizawa, 2012). It is worth mentioning that *Strategy I* species share some mechanisms for Fe and P uptake (Watt and Evans, 1999; Tomasi et al., 2009). The results relative to plant growth and physiological parameters show that NPs are excellent sources of P and Fe, and are much more effective than bulk FePO_4_, to such an extent that at times, NP-treated plants were comparable to their positive controls (where the nutrients were readily available in the growth solution). However, the responses appeared to be species-specific. Cucumber −P+NPs plants had a better P status than those treated with bulk, as reflected by their lower leaf SPAD index (Calderón-Vázquez et al., 2011; Henry et al., 2012), less stunted shoot growth (Ciereszko et al., 2002; Hawkesford et al., 2012) and higher P concentration in shoot and root tissues (**Figure 1**, **Table 1**). A similar response pattern was displayed by **-**P**-**Fe+NPs plants (**Figure 1**, **Table 1**). On the other hand, an analysis of the growth parameters of maize (**Figures 2**, **3**) suggests that in this case, both NPs and bulk are effective sources of P.

The fact that NPs are a more efficient P source for cucumber plants is also confirmed by their total P content in the shoots: only this species displayed significantly greater levels of this nutrient after treatment with NPs (**Supplementary Tables S1**, **S2**).

As regards Fe nutrition, significant differences between NP- and bulk treatments were only evident in maize, as reflected by SPAD index values, shoot development, shoot Fe concentration and total quantity of this nutrient (**Figure 2**, **Table 2**, **Supplementary Table S2**). It can be reasonably inferred that the phytosiderophore-based strategy benefits more from the presences of nano-sized FePO_4_ than that relying on acidification and reduction.

Positive effects brought about by applications of P-containing NPs on plant growth and physiology have been described in soybean (Liu and Lal, 2014), peanut (Hegab et al., 2018) and rice (Miranda-Villagómez et al., 2019b). In these investigations however the effectiveness of these compounds was appraised against non-nanomaterials having a different chemical composition from that of the NPs used (Liu and Lal, 2014; Miranda-Villagómez et al., 2019a; Miranda-Villagómez et al., 2019b). The same is true for Fe, given that the literature available mostly compares the effects of Fe-containing NPs with other forms of the micronutrient such as FeCl_3_ and Fe(III)-EDTA (Ghafariyan et al., 2013; Rui et al., 2016; Hu et al., 2017; Palmqvist et al., 2017; Yuan et al., 2018). Our investigation on the other hand compares two forms (nano- and non-nano-scale) of the same salt, and the results support the idea that nutrient availability is increased when supplied in sub-micron particles. The different responses displayed by the two species may be linked to specific mechanisms possessed by cucumber and maize roots triggered by low Fe and, in part, P bioavailability. The physicochemical properties of nanomaterials can in fact be modified by their interaction with compounds present in the environment and metabolites extruded by the roots (Zulfiqar et al., 2019). In our experimental system, the NPs suspended in the nutrient solution could easily reach, accumulate, and interact with the root surface. The release of root exudates (*e.g.* organic acids, phenolic compounds such as reducing agents and phytosiderophores) and the acidification of the rhizosphere by the plasma membrane H^+^−ATPase can increase the bioavailability of both P and Fe (Chiou and Lin, 2011; Kobayashi and Nishizawa, 2012). Our experiments show that in cucumber plants, NPs are more effective than the bulk counterpart in boosting the uptake of P, a macronutrient obviously required in greater amounts than Fe. We also observed a strong orange staining of the roots when NPs were used as the source of P (hence, both **-**P+NPs and **-**P**-**Fe+NPs plants). Root activities such as the release of exudates and rhizosphere acidification may contribute to modifying the FePO_4_ NPs accumulating at the cucumber root surface (Otani and Ae, 1996; Tsai and Schmidt, 2017a; Tsai and Schmidt, 2017b). Organic acids might dissolve the FePO_4_ NPs by Fe chelation reactions, thus releasing PO_4_^3−^, which is then taken up by the roots. Meanwhile, the excess Fe present at the rhizosphere could form iron-oxyhydroxyde deposits at the root surface. It has been reported that the organic ligands (organic acids and siderophores) released by plants and microorganisms promote the formation of ferrihydrite (Violante et al., 2003). This process may occur in the roots of cucumber plants having NPs as the source of P (**-**P+NPs and **-**P**-**Fe+NPs) by virtue of the great mobility and surface/volume ratio of these particles, as suggested by TEM analysis of the tertiary roots displaying an orange staining (**Supplementary Figure S7**). Only in proximity of the cell wall of NP-treated roots did we observe predominantly spherical, electron-dense structures, similar in shape to ferrihydrite (**Figures 4F, H**) (Violante et al., 2003). Some however exhibited a lath-like shape (**Figures 4F, H**) typical of goethite, whose production by the conversion of ferrihydrite occurs in acidic environments (Vodyanitskii, 2010). In addition, ESAM−EDAX analyses confirmed the presence excess of Fe on the root surface of the cucumber plants treated with NPs as source of P (**-**P+NPs and **-**P**-**Fe+NPs). This result was unsurprising, given the higher values of the Fe/P ratio (**Supplementary Data set S1**, **Supplementary Data set S3**). The literature reports that 16 plant species belonging to 11 different families were able to form orange Fe plaques at the root surface – consisting in agglomerated iron-oxyhydroxide NPs – when exposed to high concentrations of ionic Fe (i.e. ≥ 0.1 mM) (Pardha-Saradhi et al., 2014). It can be presumed that when cucumber plants are treated with NPs, their root metabolic activities can dissolve these compounds, with a consequent, rapid acquisition of P. The excess Fe not taken up would then cause the formation of iron-oxyhydroxide. On the other hand, since NPs can migrate towards the root surface, they would interact with the phytosiderophores (PSs) released by maize roots more effectively than bulk FePO_4_. PSs can form stable complexes with Fe(III) (von Wirén et al., 2000), hence it would dissolve the FePO_4_ bound to the NPs and chelate the released Fe, thus preventing its precipitation. We also know that Fe(III)-PS complexes can be taken up by specific plasma membrane transporters (Kobayashi and Nishizawa, 2012). Taking this into account, it can be presumed that the interaction of NPs with PSs could lead to higher concentrations of Fe(III)-PS complexes at the rhizosphere. This would explain the higher Fe concentration and total content found in NP-treated maize plants (**Table 2**, **Supplementary Table S2**).

An interesting find is the absence of NPs inside the symplast of cucumber root cells (**Figure 4**). Several papers describe an internalization and translocation of 36- to 50-nm-large NPs (Wang et al., 2016), hence exceeding the uptake-exclusion threshold of the cell wall. The average size of the FePO_4_ NPs employed ranges between 20 to 25 nm (Sega et al., 2019). However, these particles can aggregate into structures with a mean peak at 59 nm. This could explain the lack of internalization and their ability to accumulate on the outer layer of the epidermal cell wall where they may act as nutrient reservoirs.

**Figure 6:**
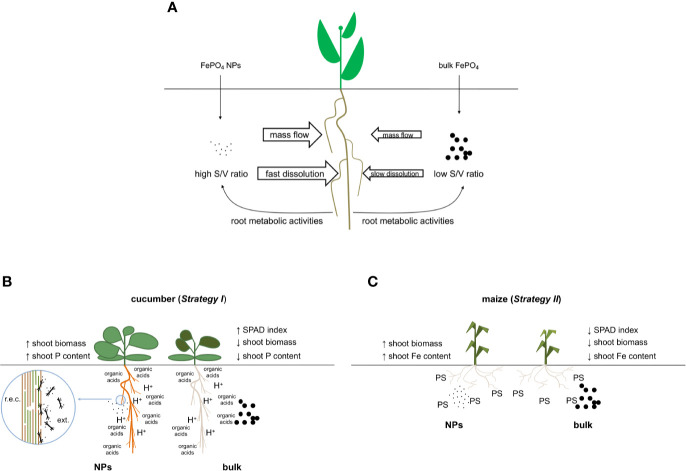
Conceptual model of the interaction between roots and FePO_4_, either as NPs or in bulk form. **(A)** Nano-sized and bulk forms in the growth solution; **(B)** details of their interaction with cucumber (*Strategy I* species) roots when used as P source and **(C)** details of their interaction with maize (*Strategy II* species) roots when used as the source of Fe; r.e.c.: root epidermal cell, ext., external side; PS, phytosiderophore.

In conclusion, our data indicate that FePO_4_ NPs are an efficient source of P and Fe, particularly when compared to their non-nano counterpart. We can speculate that thanks to their sub-micron size, larger amounts of NPs can reach the root surface of the plant. Moreover, the great surface to volume ratio of NPs and the action of the roots would ensure that they are more rapidly dissolved than bulk FePO_4_ (**Figure 6A**). The response can be affected by species-specific mechanisms (**Figures 6B, C**) leading to differences in the use of the two nutrients present in the NPs. Finally, these results can be the starting point for the development of a new class of fertilizers with a lower impact on the environment.

## Data Availability Statement

All datasets presented in this study are included in the article/**Supplementary Material**.

## Author Contributions

ZV and DS conceived the research. AZ directed the experiments. DS and BB performed the experiments. DS, AZ, BB, and ZV analyzed and interpreted the data. AZ and ZV wrote the manuscript. DS, BB, AZ, and ZV revised the manuscript. ZV obtained the funding to carry out the investigation. All authors contributed to the article and approved the submitted version.

## Conflict of Interest

The authors declare that the research was conducted in the absence of any commercial or financial relationships that could be construed as a potential conflict of interest.
