# Supplementary material for: FePO4 NPs Are an Efficient Nutritional Source for Plants: Combination of Nano-Material Properties and Metabolic Responses to Nutritional Deficiencies
Source: Front Plant Sci. 2020 Sep 30;11:586470. doi: 10.3389/fpls.2020.586470 (PMC7554371; doi:10.3389/fpls.2020.586470)
Supplement: Supplementary file 1 [file DataSheet_1.pdf]

Supplementary materials

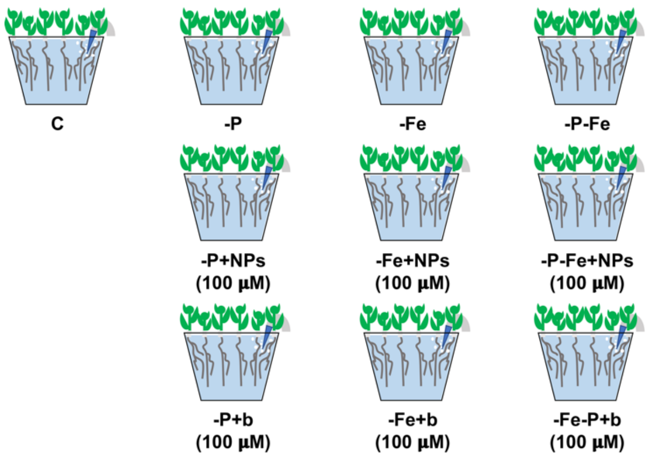

Supplementary Figure S1. Design of hydroponics experiments performed to test the ability of NPs to supply P, Fe and both nutrient to cucumber and maize plants. C: positive controls, cucumber and maize plants grown in a complete nutrient solution; -P, cucumber and maize plants grown without P; -Fe, cucumber and maize plants grown without Fe; -P-Fe, cucumber and maize plants grown without P and Fe; -P+NPs, cucumber and maize plants grown with $FePO_4$ NPs as P source; -Fe+NPs, cucumber and maize plants grown with $FePO_4$ NPs as Fe source; -P-Fe+NPs, cucumber and maize plants grown with $FePO_4$ NPs as P and Fe source; -P+b, cucumber and maize plants grown with bulk $FePO_4$ as P source; -Fe+b, cucumber and maize plants grown with bulk $FePO_4$ as Fe source; -P-Fe+b, cucumber and maize plants grown with bulk $FePO_4$ as P and Fe source.

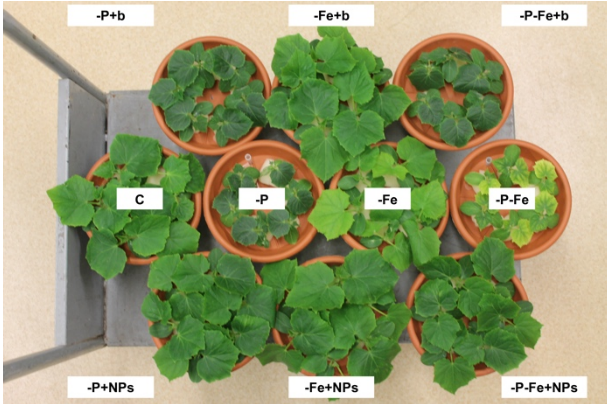

Supplementary Figure S2. Shoots of cucumber plants at the end of the treatment (14 days). C: positive controls, *i.e.* plants grown in a complete nutrient solution; -P, plants grown in the absence of P; -Fe, in the absence of Fe; -P-Fe, in the absence of both P and Fe; -P+NPs, plants grown with $FePO_4$ NPs as P source; -Fe+NPs, with $FePO_4$ NPs as Fe source; -P-Fe+NPs, with $FePO_4$ NPs as the source of both P and Fe; -P+b, plants grown with bulk $FePO_4$ as P source; -Fe+b, with bulk $FePO_4$ as Fe source; -P-Fe+b, with bulk $FePO_4$ as the source of both P and Fe.

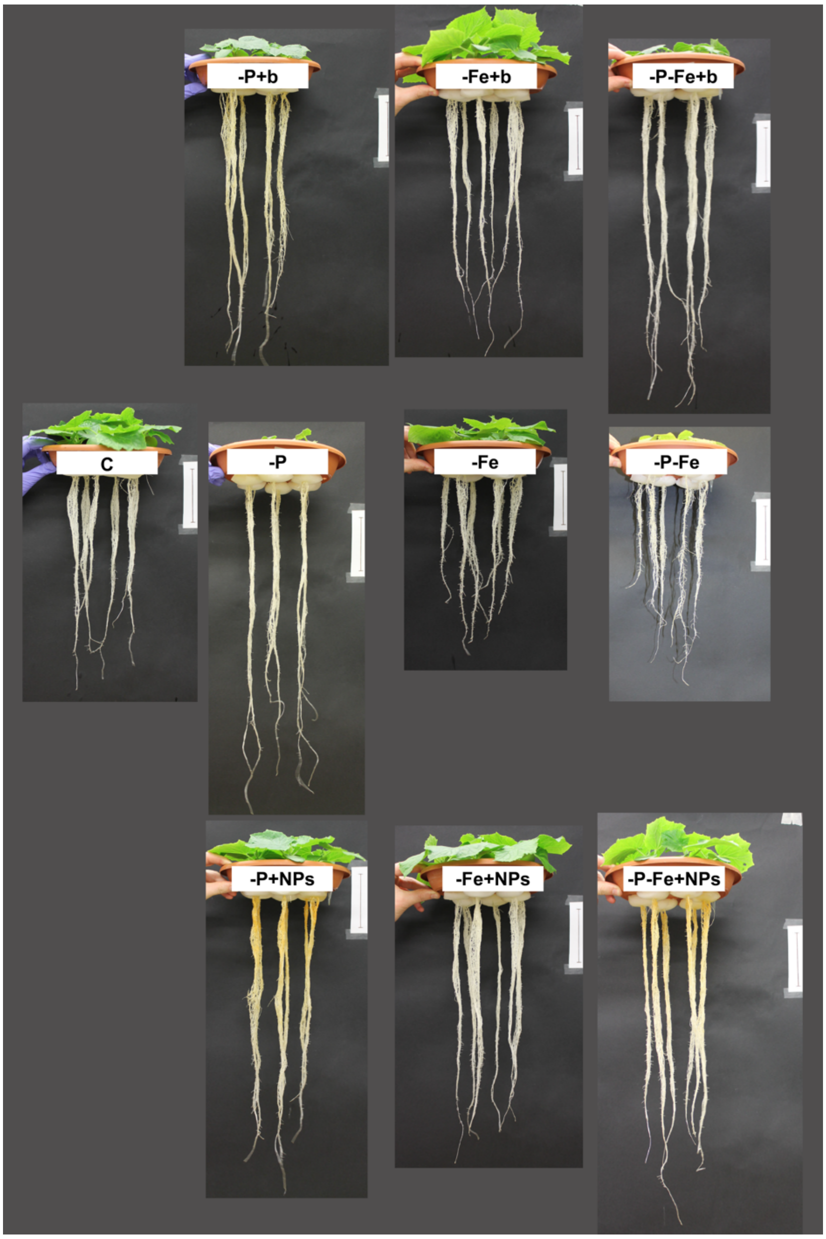

Supplementary Figure S3. Roots of cucumber plants at the end of the treatment (14 days). C: positive controls, *i.e.* plants grown in a complete nutrient solution; -P, plants grown in the absence of P; -Fe, in the absence of Fe; -P-Fe, in the absence of both P and Fe; -P+NPs, plants grown with $FePO_4$ NPs as P source; -Fe+NPs, with $FePO_4$ NPs as Fe source; -P-Fe+NPs, with $FePO_4$ NPs as the source of both P and Fe; -P+b, plants grown with bulk $FePO_4$ as P source; -Fe+b, with bulk $FePO_4$ as Fe source; -P-Fe+b, with bulk $FePO_4$ as the source of both P and Fe.

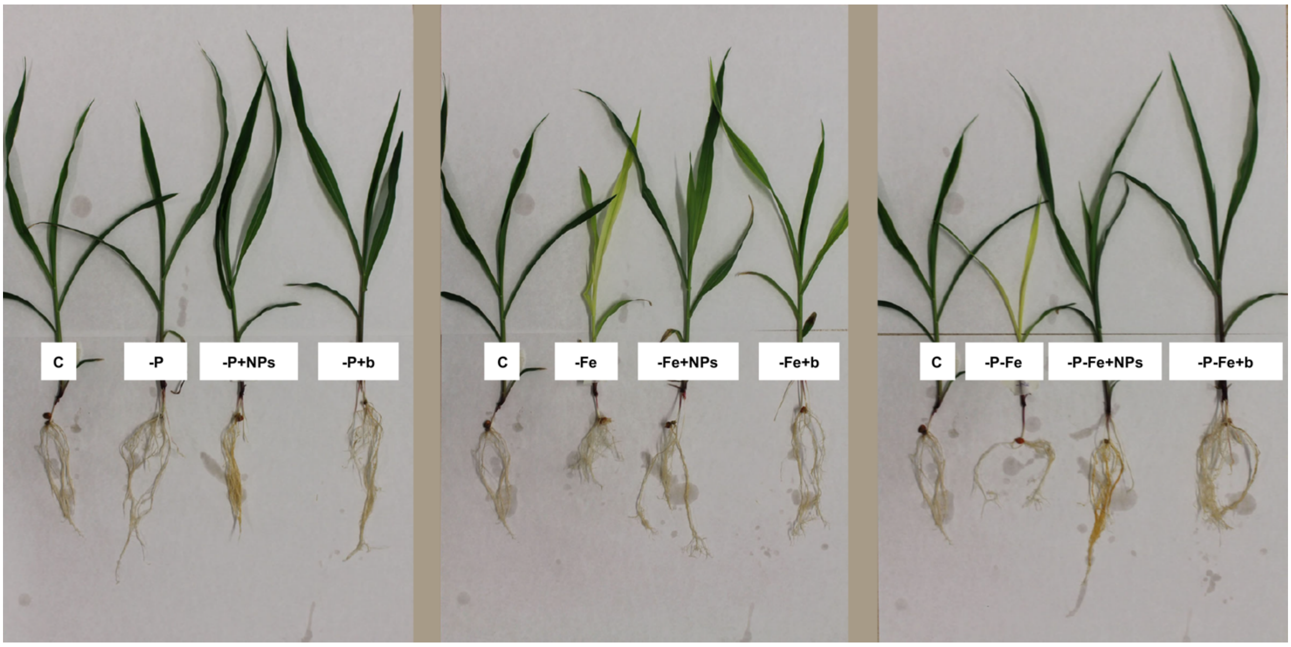

Supplementary Figure S4. Maize plants at the end of the treatment (17 days). -P, plants grown in the absence of P; -P+NPs, plants grown with $FePO_4$ NPs as P source; -P+b, with bulk $FePO_4$ as P source; -Fe, plants grown in the absence of Fe; -Fe+NPs, in the presence of $FePO_4$ NPs as Fe source; -Fe+b, in the presence of bulk $FePO_4$ as Fe source; -P-Fe, in the absence of both P and Fe; -P-Fe+NPs, with $FePO_4$ NPs as the source of both P and Fe; -P-Fe+b, with bulk $FePO_4$ as the source of P and Fe.

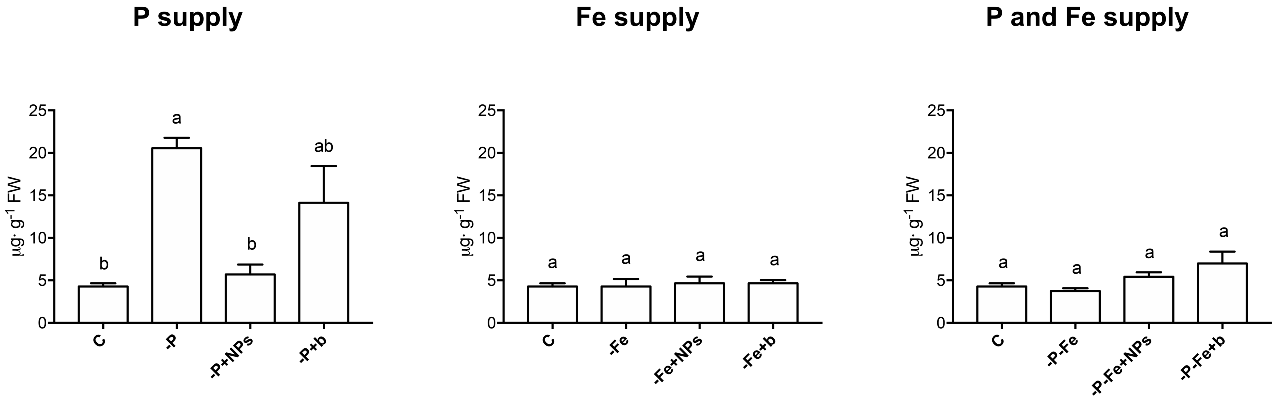

Supplementary Figure S5. Anthocyanin contents (μg g^{-1} FW) in roots of (17-day-old) maize plants, expressed as cyanidine-3-glucoside. Plants treated with NPs as the source of P, Fe or both nutrients (-P+NPs, -Fe+NPs and -P-Fe+NPs, respectively) were compared with positive controls (C), plants grown in the absence of the respective nutrients (-P, -Fe and -P-Fe) or with bulk $FePO_4$ as the source of P, Fe or both. Data are expressed as means ± *SE* (*n*= 3, three independent experiments with one plant each; one-way ANOVA with Tukey's post hoc test, $p < 0.01$, significant differences are indicated by different letters).

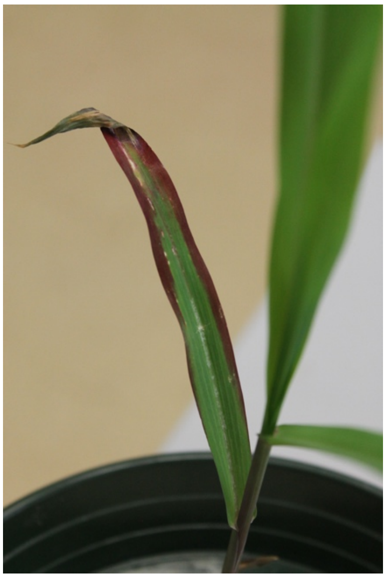

Supplementary Figure S6. Leaf symptoms in 17-days-old maize plants grown in the absence of P (-P, negative control).

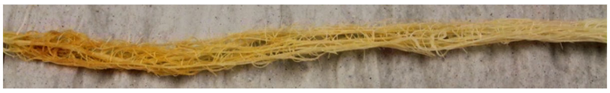

Supplementary Figure S7. Detail of the roots of a maize plant grown for 17 days in the presence of $FePO_4$ NPs as the source of P.

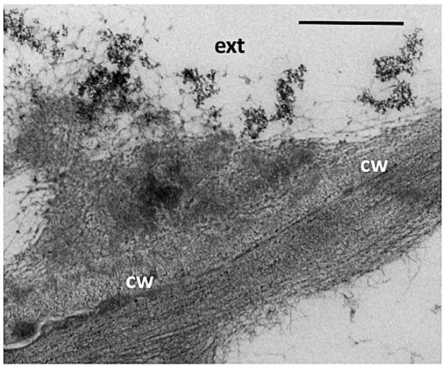

Supplementary Figure S8. TEM image of an epidermal cell wall of a tertiary root from a cucumber plant grown in the presence of $FePO_4$ NPs as the source of Fe (-Fe+NPs); cw: cell wall. Bar: 500 nm.

Supplementary Table S1. Total P and Fe contents in the shoots of cucumber plants treated for 14 days with NPs as the source of either P, Fe or both nutrients (-P+NPs, -Fe+NPs and -P-Fe+NPs), measured and compared with those of positive controls (C), plants grown in the absence of these nutrients (-P, -Fe and -P-Fe, respectively) or in the presence of bulk $FePO_4$ as the source of Fe, P or both. Data are expressed as mean ± SE (n= 9, three independent experiments with three plants each; one-way ANOVA with Tukey's post hoc test, $p < 0.05$, significant differences are indicated by different letters).

**P supply**				
	**C**	**-P**	**-P+NPs**	**-P+b**
P (mg)	3.56 ± 0.22 a	0.21 ± 0.01 c	1.20 ± 0.12 b	0.38 ± 0.02 c
Fe (μg)	46.97 ± 6.23 a	25.43 ± 3.51 b	37.09 ± 3.35 ab	39.69 ± 4.52 ab
**Fe supply**				
	**C**	**-Fe**	**-Fe+NPs**	**-Fe+b**
P (mg)	3.56 ± 0.22 a	1.53 ± 0.25 b	3.74 ± 0.24 a	3.60 ± 0.25 a
Fe (μg)	46.97 ± 6.23 a	12.30 ± 2.46 b	52.22 ± 5.89 a	42.15 ± 5.16 a
**P and Fe supply**				
	**C**	**-P-Fe**	**-P-Fe+NPs**	**-P-Fe+b**
P (mg)	3.56 ± 0.22 a	0.25 ± 0.01 c	0.89 ± 0.08 b	0.30 ± 0.01 c
Fe (μg)	46.97 ± 6.23 a	10.90 ± 1.71 c	26.06 ± 2.65 b	18.22 ± 1.90 bc

Supplementary Table S2. Total P and Fe contents in the shoots of maize plants treated for 17 days with NPs as the source of either P, Fe or both nutrients (-P+NPs, -Fe+NPs and -P-Fe+NPs), measured and compared with those of positive controls (C), plants grown in the absence of these nutrients (-P, -Fe and -P-Fe, respectively) or in the presence of bulk $FePO_4$ as the source of Fe, P or both. Data are expressed as mean ± SE (n= 9, three independent experiments with three plants each; one-way ANOVA with Tukey's post hoc test, $p < 0.05$, significant differences are indicated by different letters).

P supply				
	**C**	**-P**	**-P+NPs**	**-P+b**
P (mg)	4.88 ± 0.40 a	0.68 ± 0.09 b	1.38 ± 0.07 b	0.89 ± 0.08 b
Fe (µg)	58.86 ± 5.22 b	48.83 ± 6.95 b	103.27 ± 9.58 a	74.40 ± 10.42 ab
**Fe supply**				
	**C**	**-Fe**	**-Fe+NPs**	**-Fe+b**
P (mg)	4.88 ± 0.40 a	2.99 ± 0.27 b	4.07 ± 0.46 ab	3.42 ± 0.37 b
Fe (µg)	58.86 ± 5.22 a	9.65 ± 1.19 c	42.51 ± 3.97 b	22.05 ± 1.55 c
**P and Fe supply**				
	**C**	**-P-Fe**	**-P-Fe+NPs**	**-P-Fe+b**
P (mg)	4.88 ± 0.40 a	0.56 ± 0.06 b	1.24 ± 0.09 b	0.62 ± 0.12 b
Fe (µg)	58.86 ± 5.22 a	8.10 ± 1.29 c	44.65 ± 6.40 b	31.07 ± 3.99 c
