# Supplementary material for: FePO4 NPs Are an Efficient Nutritional Source for Plants: Combination of Nano-Material Properties and Metabolic Responses to Nutritional Deficiencies
Source: Front Plant Sci. 2020 Sep 30;11:586470. doi: 10.3389/fpls.2020.586470 (PMC7554371; doi:10.3389/fpls.2020.586470)
Supplement: Supplementary file 2 [file DataSheet_2.pdf]

Supplementary Data set S1. ESEM-EDAX analysis carried out on the roots of cucumber plants grown in the presence of $FePO_4$ NPs as P source (-P+NPs).

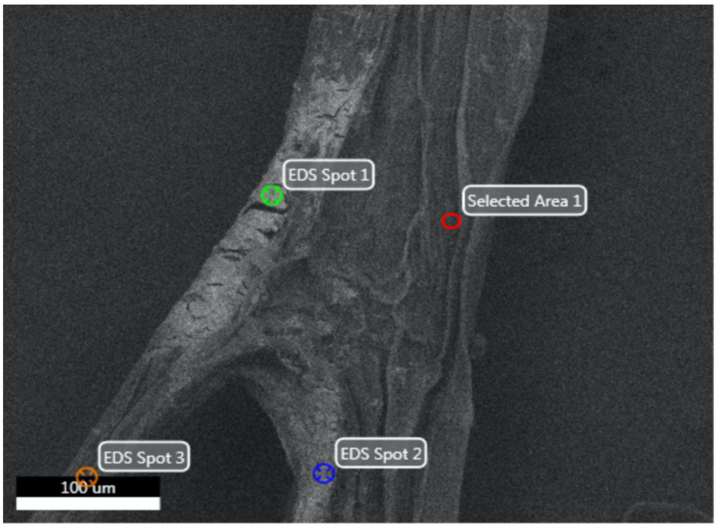

kV: 20; Mag: 300; Takeoff: 48.7; Live Time(s): 48.4; Amp Time(µs): 7.68; Resolution:(eV) 129.3

Selected Area 1

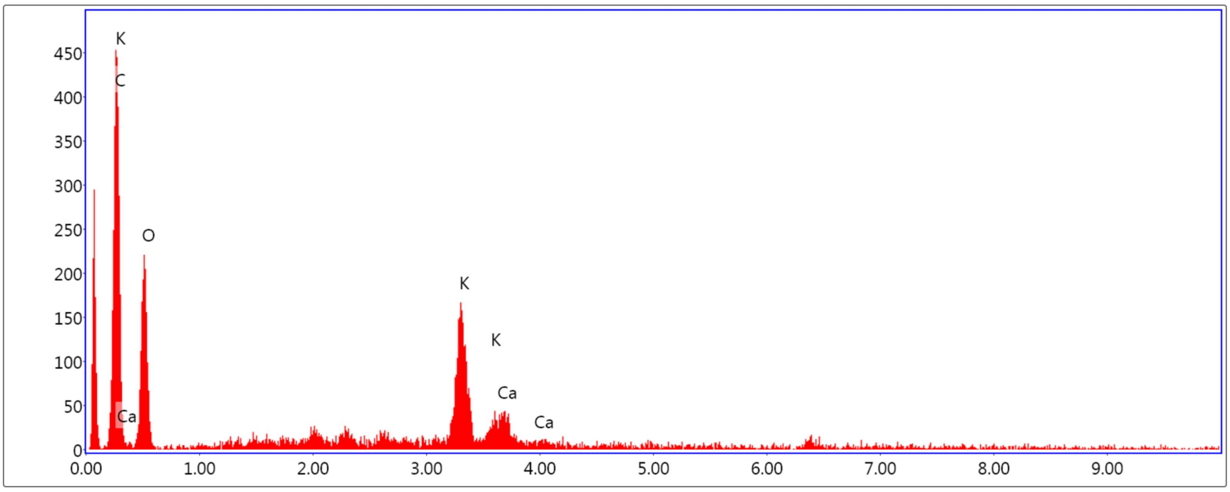

Lsec: 48.4   0 Cnts   0.000 keV   Det: Element-C2B Det

X-Ray spectrum of Selected Area 1.

eZAF Smart Quant Results of Selected Area 1

Element	Weight%	Atomic%	Net Int.	Error%	Kratio	Z	R	A	F
C K	32.79	45.15	47.88	7.75	0.2049	1.0624	0.9643	0.5879	1.0000
O K	43.37	44.83	27.78	12.63	0.0759	1.0170	0.9851	0.1721	1.0000
K K	17.46	7.38	37.11	4.80	0.1557	0.8578	1.0569	1.0119	1.0271
CaK	6.38	2.63	10.62	9.28	0.0540	0.8734	1.0612	0.9538	1.0153

kV: 20; Mag: 300; Takeoff: 48.7; Live Time(s): 48.3; Amp Time(µs): 7.68; Resolution:(eV) 129.3

EDS spot 1

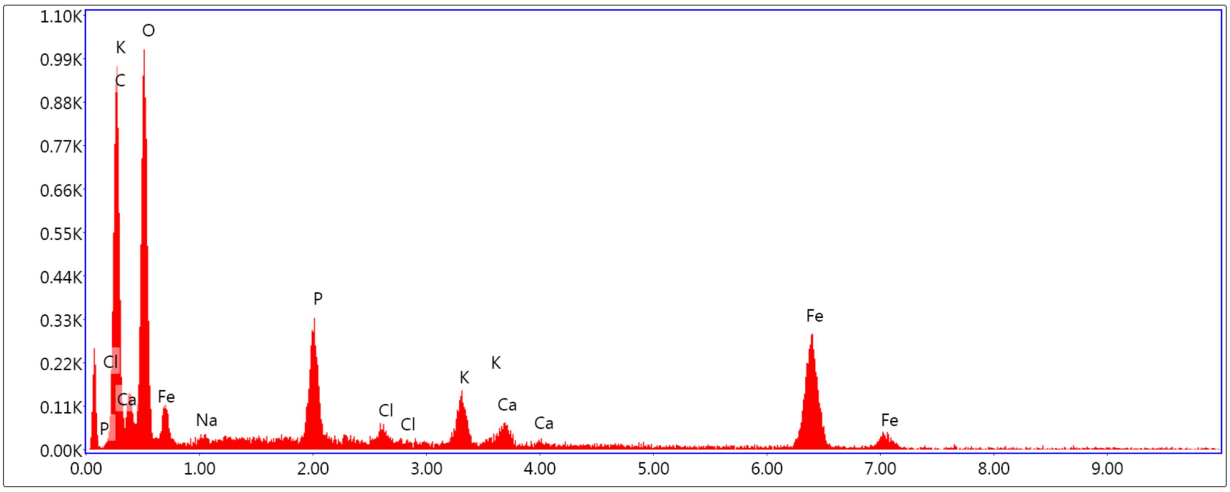

Lsec: 48.3 0 Cnts 0.000 keV Det: Element-C2B Det

X-Ray spectrum of EDS spot 1.

eZAF Smart Quant Results of EDS spot 1

Element	Weight%	Atomic%	Net Int.	Error%	Kratio	Z	R	A	F
C K	27.46	43.29	75.31	12.35	0.0886	1.1024	0.9409	0.2926	1.0000
O K	34.59	40.93	132.56	9.62	0.0996	1.0570	0.9631	0.2725	1.0000
NaK	1.03	0.85	4.05	27.10	0.0031	0.9620	0.9900	0.3142	1.0021
P K	5.66	3.46	54.48	5.71	0.0438	0.9265	1.0187	0.8250	1.0126
ClK	0.85	0.45	7.75	21.13	0.0072	0.8994	1.0308	0.9088	1.0292
K K	3.23	1.56	25.46	10.82	0.0294	0.8956	1.0416	0.9663	1.0516
CaK	1.73	0.81	11.66	17.13	0.0163	0.9122	1.0465	0.9718	1.0659
FeK	25.46	8.63	77.33	4.15	0.2173	0.8147	1.0680	1.0071	1.0403

Fe/P ratio (Fe Atomic%/ P Atomic%): 2.49

kV: 20; Mag: 300; Takeoff: 48.7; Live Time(s): 48.3; Amp Time(μs): 7.68; Resolution:(eV) 129.3

EDS spot 2

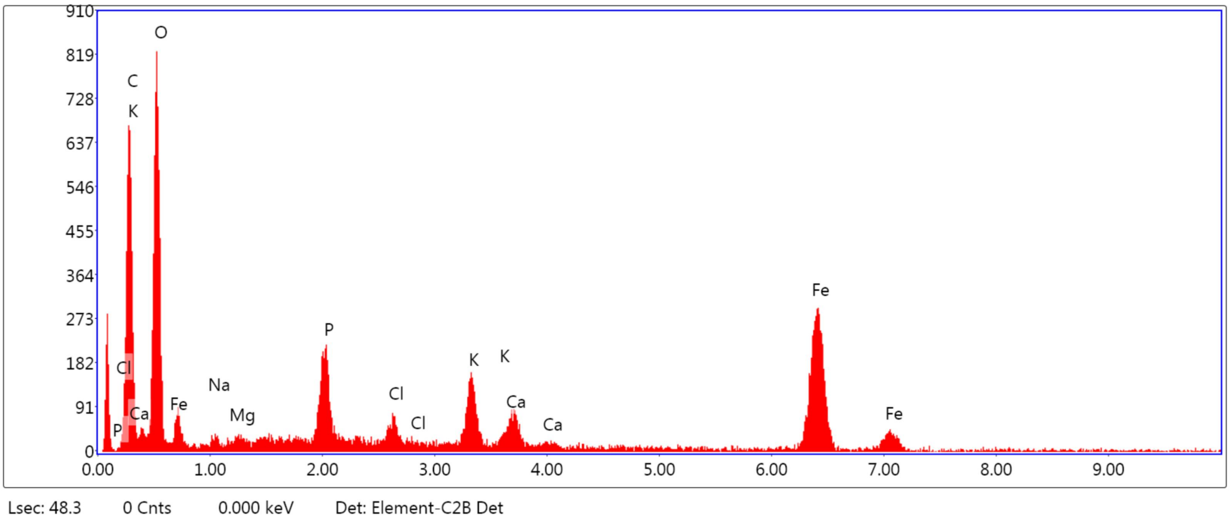

X-Ray spectrum of EDS spot 2.

eZAF Smart Quant Results of EDS spot 2

Element	Weight%	Atomic%	Net Int.	Error%	Kratio	Z	R	A	F
C K	23.10	39.94	54.24	11.50	0.0723	1.1243	0.9283	0.2784	1.0000
O K	30.19	39.19	103.63	9.82	0.0882	1.0788	0.9512	0.2709	1.0000
NaK	0.90	0.82	2.98	33.04	0.0026	0.9825	0.9790	0.2930	1.0020
MgK	0.42	0.36	2.50	44.35	0.0018	1.0000	0.9871	0.4336	1.0036
P K	4.64	3.11	39.19	6.55	0.0357	0.9470	1.0088	0.8011	1.0137
ClK	1.42	0.83	11.52	18.12	0.0121	0.9195	1.0215	0.8995	1.0312
K K	4.42	2.35	31.28	8.47	0.0409	0.9158	1.0328	0.9585	1.0536
CaK	2.83	1.47	17.10	11.59	0.0271	0.9329	1.0380	0.9616	1.0657
FeK	32.08	11.93	87.39	3.67	0.2782	0.8338	1.0614	1.0036	1.0361

Fe/P ratio (Fe Atomic%/ P Atomic%): 3.84

kV: 20; Mag: 300; Takeoff: 48.7; Live Time(s): 48.6; Amp Time(µs): 7.68; Resolution:(eV) 129.3

EDS spot 3

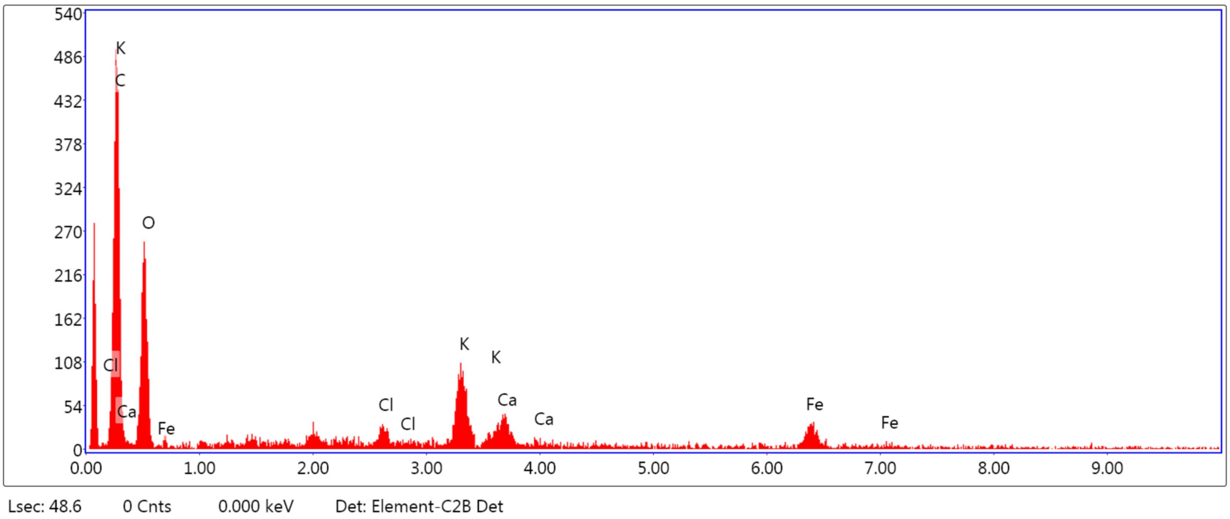

X-Ray spectrum of EDS spot 3.

eZAF Smart Quant Results of EDS spot 3

Element	Weight%	Atomic%	Net Int.	Error%	Kratio	Z	R	A	F
C K	38.44	52.53	52.13	8.87	0.1906	1.0656	0.9613	0.4654	1.0000
O K	37.59	38.56	32.04	12.17	0.0748	1.0204	0.9823	0.1952	1.0000
ClK	1.39	0.64	4.24	26.87	0.0122	0.8654	1.0452	0.9700	1.0439
K K	8.90	3.73	22.20	6.71	0.0796	0.8614	1.0550	0.9995	1.0392
CaK	4.39	1.80	8.94	11.23	0.0388	0.8772	1.0594	0.9756	1.0339
FeK	9.30	2.73	8.87	11.61	0.0774	0.7824	1.0779	1.0054	1.0586

Fe/P ratio (Fe Atomic%/ P Atomic%): not determined, only Fe was detected.
