# Supplementary material for: FePO4 NPs Are an Efficient Nutritional Source for Plants: Combination of Nano-Material Properties and Metabolic Responses to Nutritional Deficiencies
Source: Front Plant Sci. 2020 Sep 30;11:586470. doi: 10.3389/fpls.2020.586470 (PMC7554371; doi:10.3389/fpls.2020.586470)
Supplement: Supplementary file 3 [file DataSheet_3.pdf]

Supplementary Data set S2. ESEM-EDAX analysis carried out on the roots of cucumber plants grown in the presence of $FePO_4$ NPs as Fe source (-Fe+NPs).

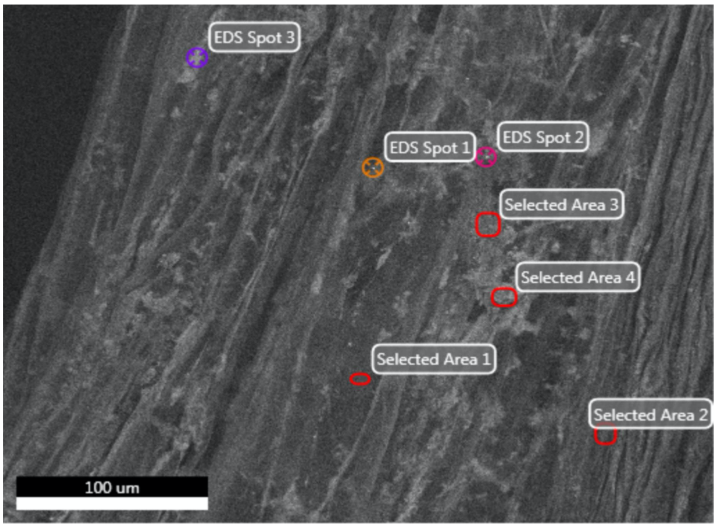

kV: 20; Mag: 400; Takeoff: 44.8; Live Time(s): 48.4; Amp Time(µs): 7.68; Resolution:(eV) 129.3

Selected Area 1

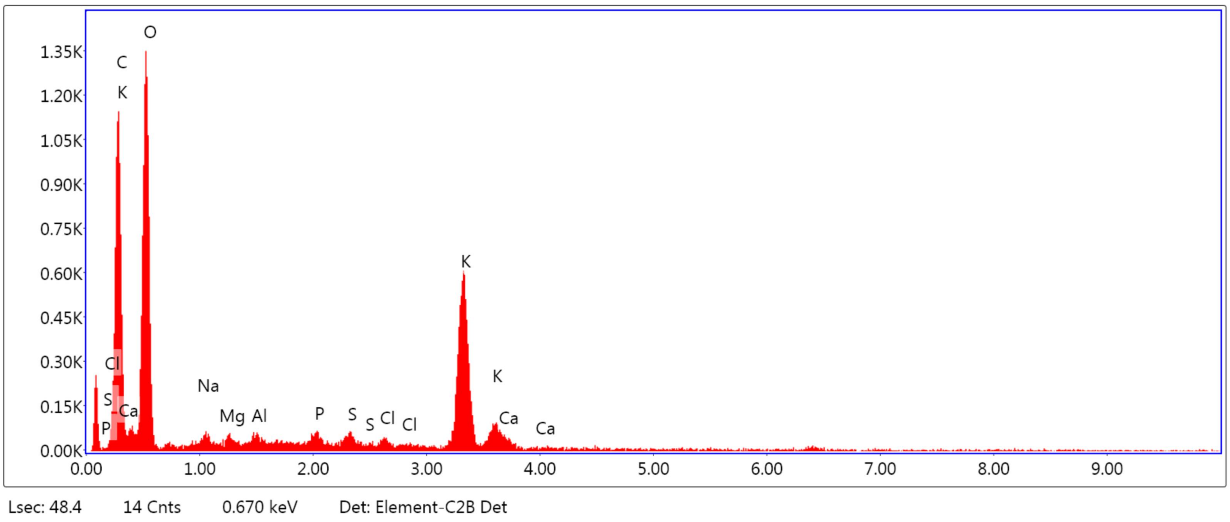

X-Ray spectrum of Selected Area 1.

eZAF Smart Quant Results of Selected Area 1

Element	Weight%	Atomic%	Net Int.	Error%	Kratio	Z	R	A	F
C K	24.25	33.41	105.18	9.07	0.1132	1.0611	0.9680	0.4400	1.0000
O K	55.21	57.11	185.86	9.89	0.1252	1.0156	0.9885	0.2233	1.0000
NaK	1.41	1.01	7.14	18.82	0.0049	0.9224	1.0131	0.3741	1.0029
MgK	0.50	0.34	4.43	29.53	0.0025	0.9381	1.0202	0.5316	1.0051
AlK	0.45	0.27	4.75	31.19	0.0028	0.9034	1.0269	0.6783	1.0088
P K	0.72	0.38	8.12	18.36	0.0057	0.8867	1.0390	0.8788	1.0234
S K	0.72	0.37	8.46	18.52	0.0062	0.9041	1.0445	0.9305	1.0351
ClK	0.41	0.19	4.40	33.03	0.0036	0.8600	1.0497	0.9629	1.0542
K K	15.02	6.36	130.12	2.86	0.1308	0.8558	1.0592	1.0010	1.0174
CaK	1.33	0.55	9.21	16.59	0.0112	0.8714	1.0634	0.9522	1.0185

Fe/P ratio (Fe Atomic%/ P Atomic%): not determined, only P was detected.

kV: 20; Mag: 400; Takeoff: 44.8; Live Time(s): 48.2; Amp Time(µs): 7.68; Resolution:(eV) 129.3

Selected Area 2

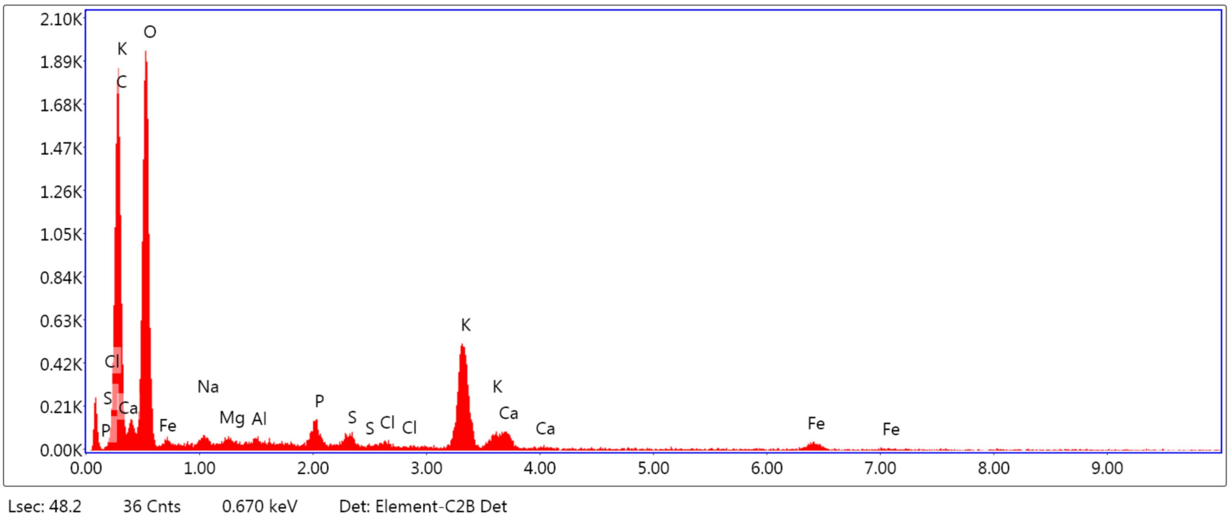

X-Ray spectrum of Selected Area 2.

eZAF Smart Quant Results of Selected Area 2

Element	Weight%	Atomic%	Net Int.	Error%	Kratio	Z	R	A	F
C K	27.41	37.06	155.85	9.44	0.1178	1.0581	0.9694	0.4062	1.0000
O K	53.49	54.29	271.69	9.56	0.1285	1.0126	0.9898	0.2374	1.0000
NaK	1.66	1.17	11.90	14.33	0.0057	0.9196	1.0144	0.3726	1.0026
MgK	0.72	0.48	8.96	15.80	0.0036	0.9353	1.0214	0.5278	1.0044
AlK	0.47	0.28	6.98	19.39	0.0028	0.9006	1.0280	0.6723	1.0076
P K	1.44	0.76	23.02	9.94	0.0114	0.8839	1.0400	0.8746	1.0184
S K	0.81	0.41	13.43	13.27	0.0069	0.9013	1.0455	0.9222	1.0268
ClK	0.40	0.18	5.99	25.42	0.0034	0.8573	1.0507	0.9559	1.0408
K K	9.65	4.01	119.10	3.12	0.0841	0.8531	1.0601	0.9971	1.0245
CaK	1.85	0.75	18.64	12.68	0.0159	0.8686	1.0643	0.9698	1.0248
FeK	2.10	0.61	10.44	16.61	0.0179	0.7742	1.0815	1.0077	1.0928

Fe/P ratio (Fe Atomic%/ P Atomic%): 0.80

kV: 20; Mag: 400; Takeoff: 44.8; Live Time(s): 48.4; Amp Time(µs): 7.68; Resolution:(eV) 129.3

Selected Area 3

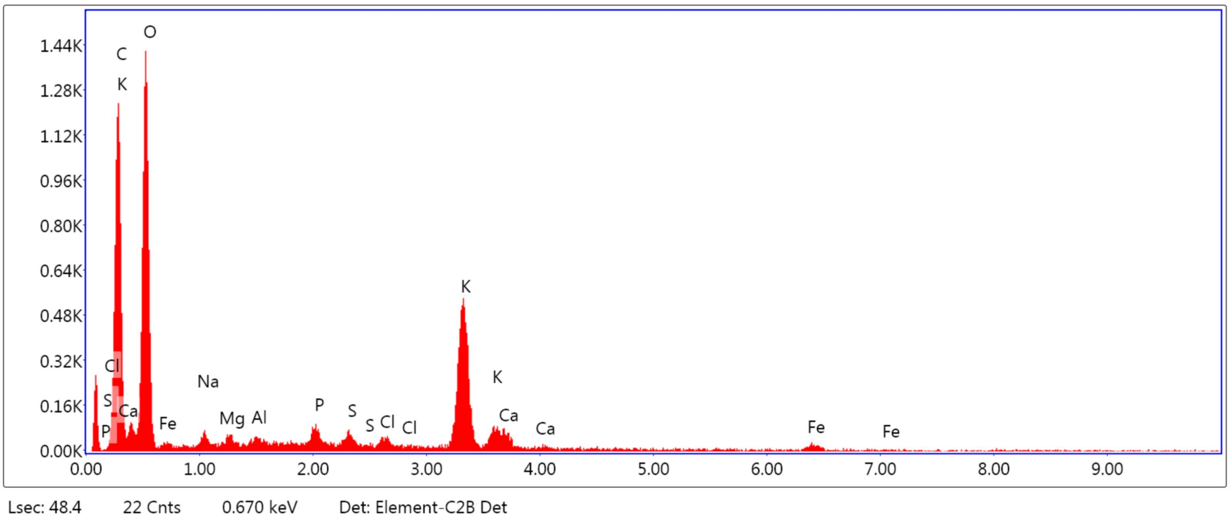

X-Ray spectrum of Selected Area 3.

eZAF Smart Quant Results of Selected Area 3

Element	Weight%	Atomic%	Net Int.	Error%	Kratio	Z	R	A	F
C K	25.52	35.14	109.80	9.17	0.1160	1.0617	0.9672	0.4280	1.0000
O K	53.80	55.61	185.60	9.89	0.1227	1.0161	0.9877	0.2244	1.0000
NaK	1.11	0.80	5.67	22.44	0.0038	0.9230	1.0125	0.3692	1.0027
MgK	0.52	0.36	4.69	29.42	0.0026	0.9388	1.0196	0.5282	1.0048
AlK	0.31	0.19	3.35	40.82	0.0019	0.9040	1.0263	0.6747	1.0084
P K	1.00	0.53	11.47	14.21	0.0079	0.8873	1.0384	0.8772	1.0216
S K	0.67	0.34	7.99	16.60	0.0058	0.9047	1.0440	0.9273	1.0324
ClK	0.53	0.25	5.80	24.66	0.0046	0.8607	1.0492	0.9607	1.0494
K K	13.23	5.59	117.12	3.09	0.1156	0.8565	1.0587	0.9992	1.0209
CaK	1.80	0.74	12.84	15.56	0.0154	0.8721	1.0629	0.9576	1.0213
FeK	1.52	0.45	5.39	23.20	0.0129	0.7774	1.0805	1.0050	1.0895

Fe/P ratio (Fe Atomic%/ P Atomic%): 0.85

kV: 20; Mag: 400; Takeoff: 44.8; Live Time(s): 48.1; Amp Time(μs): 7.68; Resolution:(eV) 129.3

Selected Area 4

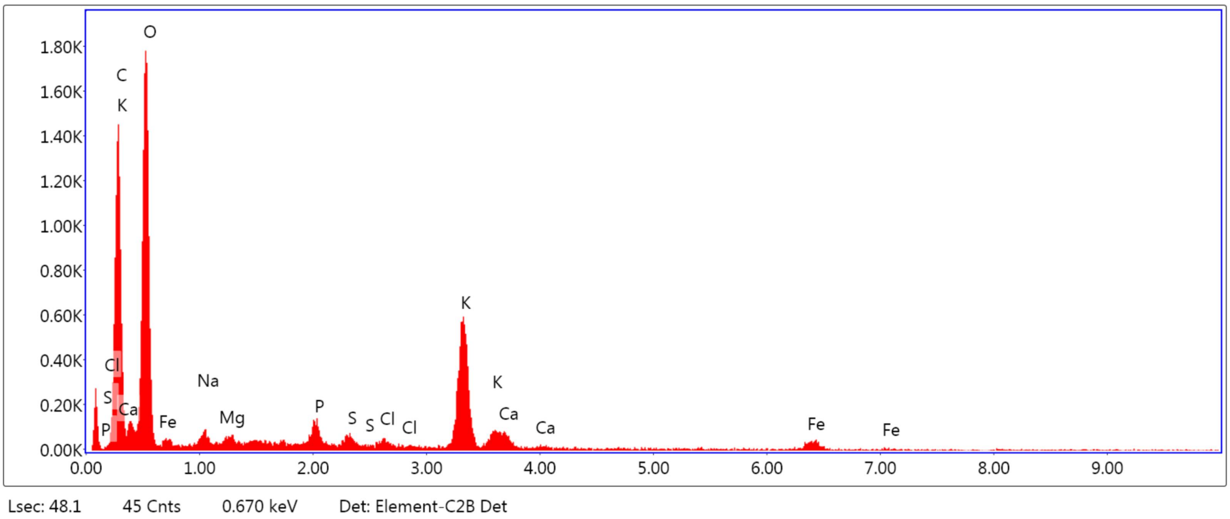

X-Ray spectrum of Selected Area 4.

eZAF Smart Quant Results of Selected Area 4

Element	Weight%	Atomic%	Net Int.	Error%	Kratio	Z	R	A	F
C K	23.18	32.45	112.80	10.05	0.0962	1.0655	0.9654	0.3897	1.0000
O K	54.22	56.99	249.67	9.56	0.1333	1.0200	0.9861	0.2411	1.0000
NaK	2.27	1.66	14.21	13.97	0.0077	0.9266	1.0110	0.3647	1.0026
MgK	1.04	0.72	11.23	15.27	0.0050	0.9424	1.0182	0.5142	1.0044
P K	1.58	0.86	22.36	10.27	0.0125	0.8909	1.0371	0.8665	1.0193
S K	0.74	0.39	10.87	15.17	0.0063	0.9084	1.0427	0.9151	1.0286
ClK	0.59	0.28	7.90	22.07	0.0051	0.8641	1.0480	0.9509	1.0433
K K	11.77	5.06	129.06	2.95	0.1029	0.8599	1.0576	0.9935	1.0228
CaK	1.75	0.73	15.55	11.16	0.0150	0.8756	1.0619	0.9591	1.0241
FeK	2.87	0.86	12.59	15.91	0.0244	0.7806	1.0798	1.0054	1.0833

Fe/P ratio (Fe Atomic%/ P Atomic%): 1.00

kV: 20; Mag: 400; Takeoff: 44.8; Live Time(s): 48.5; Amp Time(µs): 7.68; Resolution:(eV) 129.3

EDS spot 1

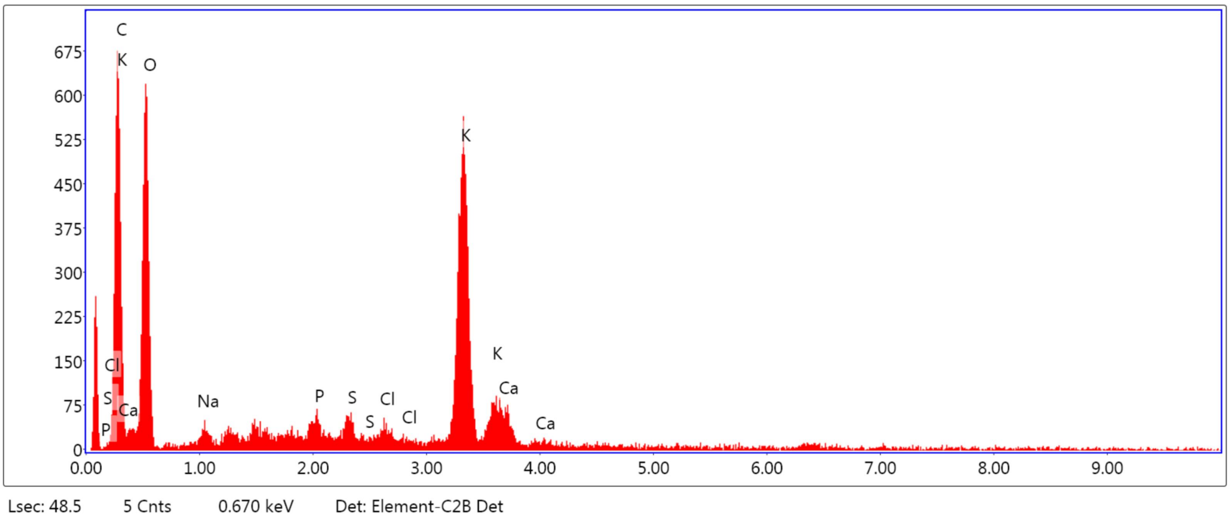

X-Ray spectrum of EDS spot 1.

eZAF Smart Quant Results of EDS spot 1

Element	Weight%	Atomic%	Net Int.	Error%	Kratio	Z	R	A	F
C K	21.24	31.26	54.54	9.66	0.1022	1.0753	0.9588	0.4474	1.0000
O K	50.18	55.43	81.21	10.89	0.0952	1.0297	0.9800	0.1843	1.0000
NaK	0.66	0.50	1.92	71.05	0.0023	0.9359	1.0055	0.3693	1.0033
P K	0.92	0.52	6.12	20.19	0.0075	0.9001	1.0323	0.8832	1.0295
S K	0.85	0.47	5.92	20.87	0.0076	0.9179	1.0381	0.9320	1.0449
ClK	0.59	0.29	3.75	33.04	0.0053	0.8732	1.0435	0.9627	1.0695
K K	22.53	10.19	113.75	3.21	0.1992	0.8691	1.0534	0.9998	1.0169
CaK	3.04	1.34	11.89	14.29	0.0252	0.8850	1.0579	0.9236	1.0148

Fe/P ratio (Fe Atomic%/ P Atomic%): not determined, only P was detected.

kV: 20; Mag: 400; Takeoff: 44.8; Live Time(s): 48.4; Amp Time(μs): 7.68; Resolution:(eV) 129.3

EDS spot 2

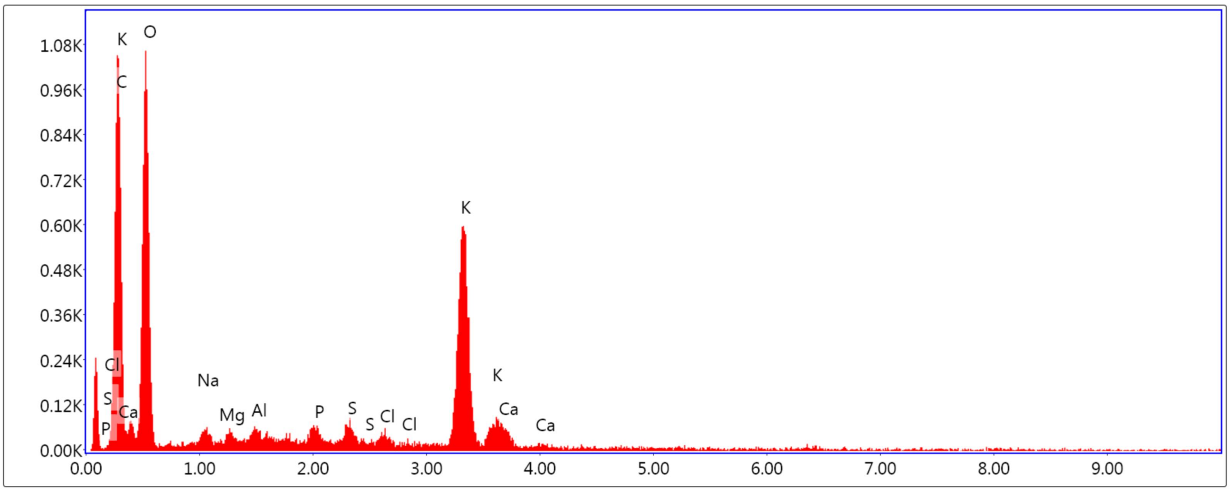

X-Ray spectrum of EDS spot 2.

eZAF Smart Quant Results of EDS spot 2

Element	Weight%	Atomic%	Net Int.	Error%	Kratio	Z	R	A	F
C K	25.42	35.59	95.70	9.37	0.1134	1.0653	0.9653	0.4188	1.0000
O K	50.55	53.13	140.54	10.28	0.1042	1.0197	0.9860	0.2022	1.0000
NaK	1.53	1.12	7.22	18.70	0.0054	0.9264	1.0109	0.3812	1.0031
MgK	0.56	0.38	4.55	25.88	0.0028	0.9422	1.0181	0.5379	1.0055
AlK	0.67	0.42	6.61	21.03	0.0042	0.9073	1.0248	0.6830	1.0094
P K	0.92	0.50	9.53	14.48	0.0074	0.8906	1.0370	0.8796	1.0248
ClK	0.65	0.31	6.36	24.81	0.0057	0.8639	1.0479	0.9601	1.0560
K K	16.93	7.28	133.49	2.82	0.1478	0.8597	1.0575	0.9982	1.0170
CaK	1.79	0.75	11.18	18.71	0.0150	0.8754	1.0618	0.9430	1.0171

Fe/P ratio (Fe Atomic%/ P Atomic%): not determined, only P was detected.

kV: 20; Mag: 400; Takeoff: 44.8; Live Time(s): 48.4; Amp Time(µs): 7.68; Resolution:(eV) 129.3

EDS spot 3

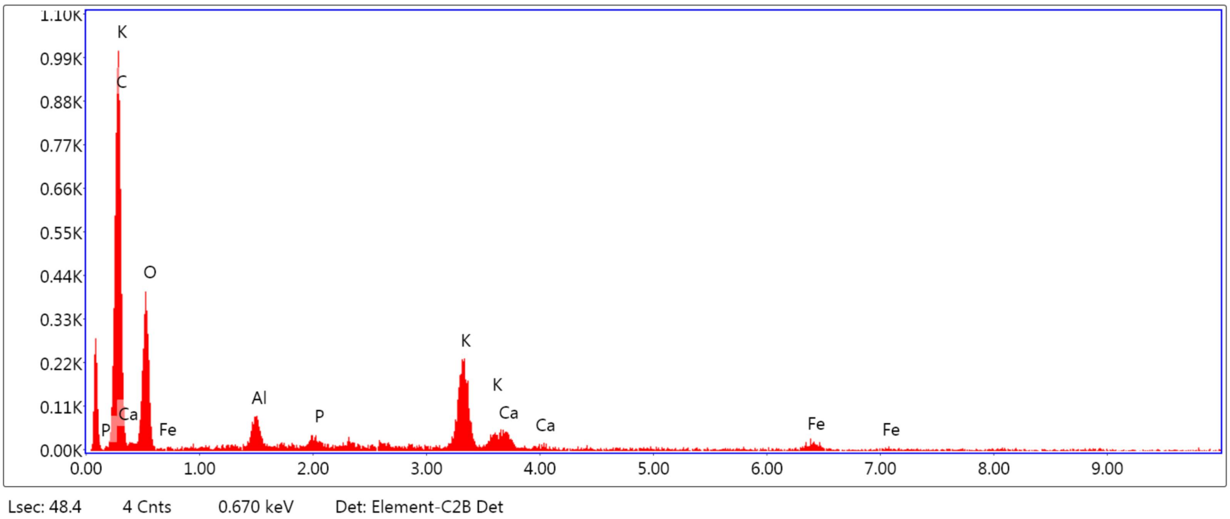

X-Ray spectrum of EDS spot 3.

eZAF Smart Quant Results of EDS spot 3

Element	Weight%	Atomic%	Net Int.	Error%	Kratio	Z	R	A	F
C K	40.44	53.14	95.10	7.78	0.2152	1.0553	0.9689	0.5041	1.0000
O K	39.21	38.68	50.31	11.73	0.0713	1.0099	0.9893	0.1799	1.0000
AlK	1.97	1.15	10.36	13.93	0.0126	0.8985	1.0276	0.7076	1.0078
P K	0.54	0.28	2.92	40.36	0.0043	0.8819	1.0396	0.8853	1.0208
K K	11.71	4.73	48.79	4.99	0.1031	0.8513	1.0597	1.0074	1.0269
CaK	2.65	1.04	8.91	18.03	0.0228	0.8668	1.0639	0.9690	1.0252
FeK	3.49	0.99	5.70	22.33	0.0293	0.7728	1.0813	1.0065	1.0790

Fe/P ratio (Fe Atomic%/ P Atomic%): 3.53
