# Supplementary material for: FePO4 NPs Are an Efficient Nutritional Source for Plants: Combination of Nano-Material Properties and Metabolic Responses to Nutritional Deficiencies
Source: Front Plant Sci. 2020 Sep 30;11:586470. doi: 10.3389/fpls.2020.586470 (PMC7554371; doi:10.3389/fpls.2020.586470)
Supplement: Supplementary file 4 [file DataSheet_4.pdf]

Supplementary Data set S3. ESEM-EDAX analysis carried out on the roots of cucumber plants grown in the presence of $FePO_4$ NPs as the source of both P and Fe (-P-Fe+NPs).

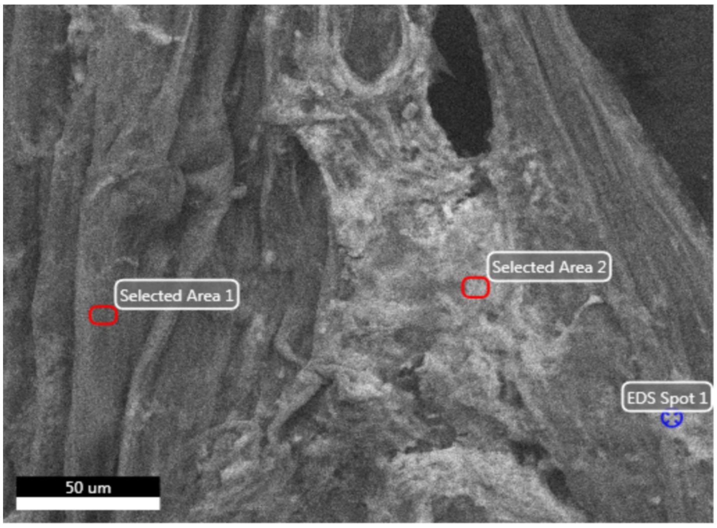

kV: 20; Mag: 600; Takeoff: 42.6; Live Time(s): 48; Amp Time(µs): 7.68; Resolution:(eV) 129.3

Selected Area 1

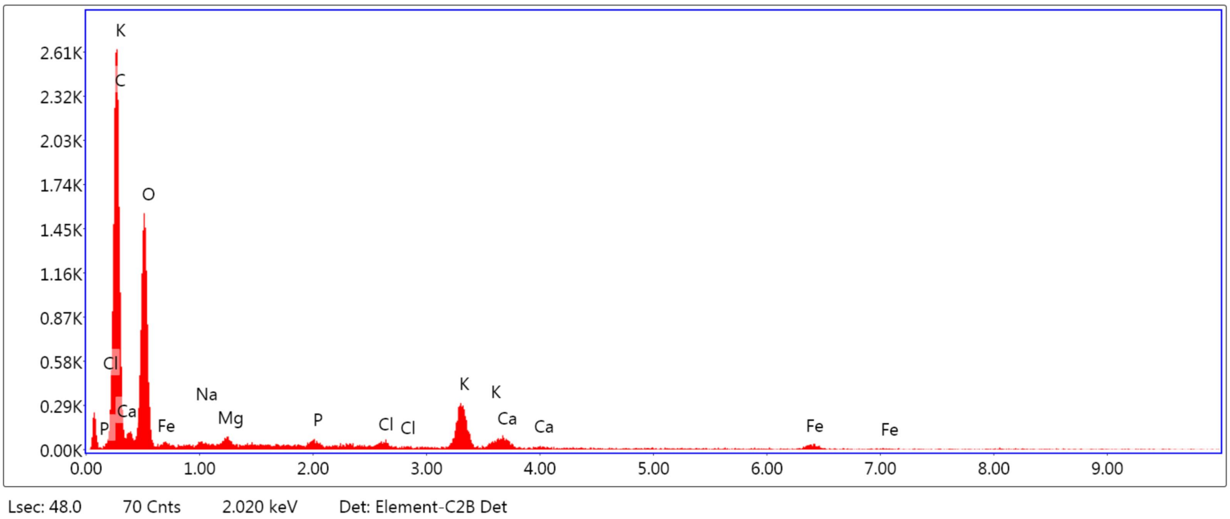

X-Ray spectrum of Selected Area 1.

eZAF Smart Quant Results of Selected Area 1

Element	Weight%	Atomic%	Net Int.	Error%	Kratio	Z	R	A	F
C K	39.59	49.53	249.49	7.70	0.1979	1.0426	0.9778	0.4794	1.0000
O K	48.30	45.37	211.14	9.91	0.1034	0.9971	0.9976	0.2147	1.0000
NaK	1.26	0.82	8.89	17.97	0.0044	0.9051	1.0213	0.3824	1.0023
MgK	0.96	0.59	11.86	13.25	0.0048	0.9204	1.0281	0.5432	1.0037
P K	0.60	0.29	9.43	14.26	0.0047	0.8696	1.0460	0.8896	1.0162
ClK	0.63	0.27	9.28	16.57	0.0054	0.8432	1.0562	0.9757	1.0363
K K	5.39	2.07	65.53	4.12	0.0470	0.8390	1.0651	1.0082	1.0312
CaK	1.63	0.61	16.43	11.14	0.0143	0.8542	1.0691	0.9940	1.0307
FeK	1.64	0.44	8.09	17.59	0.0141	0.7611	1.0851	1.0135	1.1147

Fe/P ratio (Fe Atomic%/ P Atomic%): 1.52

kV: 20; Mag: 600; Takeoff: 42.6; Live Time(s): 48.1; Amp Time(µs): 7.68; Resolution:(eV) 129.3

Selected area 2

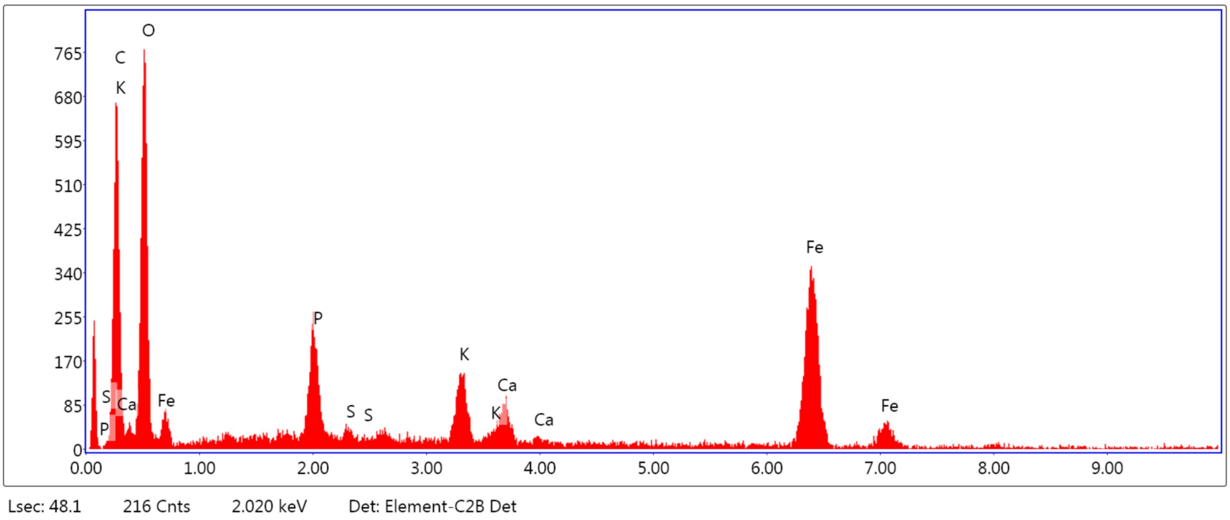

X-Ray spectrum of Selected area 2.

eZAF Smart Quant Results of Selected area 2

Element	Weight%	Atomic%	Net Int.	Error%	Kratio	Z	R	A	F
C K	23.93	41.26	58.07	11.67	0.0774	1.1241	0.9276	0.2876	1.0000
O K	30.23	39.13	102.06	9.96	0.0840	1.0786	0.9506	0.2575	1.0000
P K	4.78	3.20	42.56	8.31	0.0360	0.9470	1.0083	0.7837	1.0134
S K	0.41	0.26	3.80	31.55	0.0034	0.9661	1.0148	0.8302	1.0205
K K	4.20	2.22	32.29	7.62	0.0389	0.9159	1.0323	0.9581	1.0567
CaK	2.79	1.44	18.32	10.62	0.0267	0.9330	1.0375	0.9613	1.0699
FeK	33.67	12.49	99.87	3.54	0.2922	0.8340	1.0610	1.0048	1.0354

Fe/P ratio (Fe Atomic%/ P Atomic%): 3.90

kV: 20; Mag: 600; Takeoff: 42.6; Live Time(s): 48.3; Amp Time(µs): 7.68; Resolution:(eV) 129.3

EDS spot 1

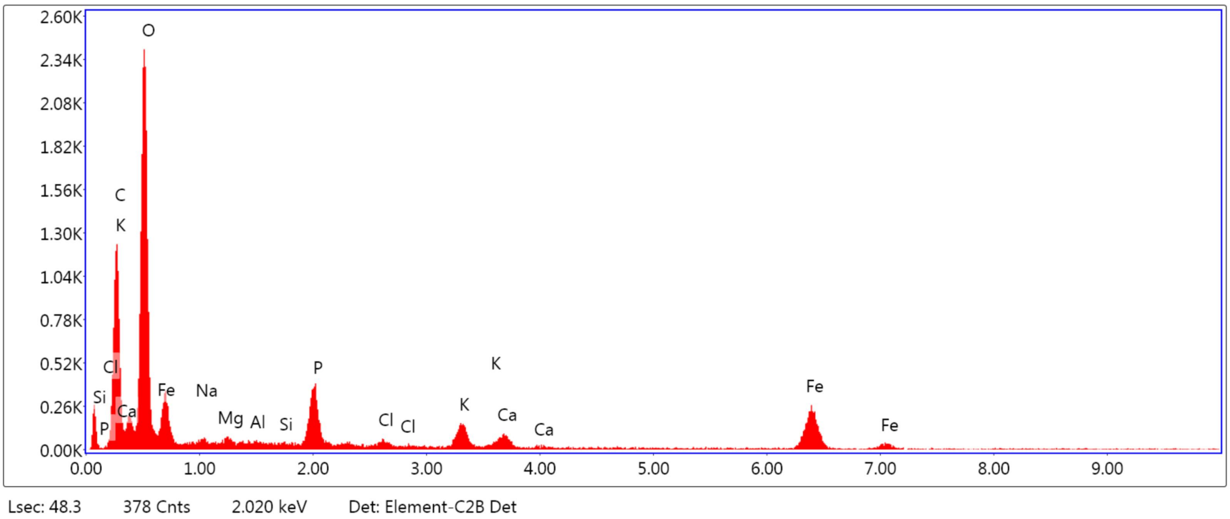

X-Ray spectrum of EDS spot 1.

eZAF Smart Quant Results of EDS spot 1

Element	Weight%	Atomic%	Net Int.	Error%	Kratio	Z	R	A	F
C K	24.27	34.82	110.99	12.26	0.0811	1.0750	0.9591	0.3110	1.0000
O K	50.18	54.05	337.61	8.78	0.1524	1.0295	0.9802	0.2950	1.0000
NaK	2.14	1.60	14.32	14.38	0.0065	0.9358	1.0057	0.3232	1.0022
MgK	0.99	0.70	11.75	15.68	0.0044	0.9519	1.0130	0.4669	1.0037
AlK	0.51	0.32	7.44	20.07	0.0029	0.9168	1.0199	0.6095	1.0063
SiK	0.24	0.15	4.16	32.53	0.0017	0.9371	1.0264	0.7330	1.0103
P K	4.24	2.36	68.95	5.23	0.0320	0.9001	1.0325	0.8289	1.0115
ClK	0.56	0.27	8.62	20.39	0.0046	0.8733	1.0437	0.9199	1.0263
K K	2.46	1.09	33.01	7.25	0.0218	0.8692	1.0536	0.9766	1.0444
CaK	1.58	0.68	18.02	13.64	0.0144	0.8852	1.0580	0.9825	1.0529
FeK	12.85	3.97	67.56	4.52	0.1084	0.7895	1.0769	1.0113	1.0569

Fe/P ratio (Fe Atomic%/ P Atomic%): 1.68
